# HURP Silencing Differentially Impacts Spindle Architecture and Metastatic Behavior in Breast Cancer Cell Lines

**DOI:** 10.3390/ijms27135897

**Published:** 2026-06-30

**Authors:** Christos Efstathiou, Stylianos Didaskalou, Lito Karkaletsou, Stella Malichetoudi, Evgenios Eftalitsidis, Andreas Girod, Maria Koffa

**Affiliations:** 1 Department of Molecular Biology and Genetics, Democritus University of Thrace, 68100 Alexandroupolis, Greece; cefstath@mbg.duth.gr (C.E.); lkarkale@mbg.duth.gr (L.K.); malichstella2016@gmail.com (S.M.);; 2 Department of Life Sciences and Medicine, University of Luxembourg, 4365 Esch-sur-Alzette, Luxembourg; andreas.girod@uni.lu

**Keywords:** HURP, TPX2, Aurora-A, NuMA, breast cancer, mitosis, spindle angle, spindle architecture, metastatic behavior

## Abstract

Chromosomal instability (CIN) arising from mitotic errors is a hallmark of cancer progression, yet how specific spindle assembly factors are co-opted to support aggressive tumor phenotypes remains incompletely understood. Hepatoma Upregulated Protein (HURP/DLGAP5), a Ran-regulated microtubule-associated protein essential for kinetochore fiber stabilization and chromosome congression, is frequently overexpressed in aggressive cancers. Here, we investigated HURP’s role across a breast cancer metastatic gradient—immortalized MCF10A, the low-metastatic luminal T47D, and the highly metastatic triple-negative MDA-MB-231 cell lines—integrating quantitative spindle analysis, kinetochore tension measurements, spindle checkpoint profiling, migration dynamics, and three-dimensional spheroid modeling. We show that total HURP protein levels increase with metastatic potential, yet spindle-bound HURP is paradoxically reduced in MDA-MB-231 cells, indicating cytoplasmic mislocalization despite increased total protein levels. HURP silencing induced cell-line-specific defects: moderate disorganization and misorientation in MCF10A and T47D cells, but catastrophic spindle collapse, apoptosis, and G2/M arrest in MDA-MB-231 cells. Mechanistically, HURP depletion disrupted the spindle-associated levels and distributions of TPX2, Aurora-A, and NuMA in a subtype-dependent manner, implicating HURP as a context-dependent stabilizer of this mitotic regulatory axis. HURP loss reduced interkinetochore tension in all cell lines, but only MCF10A and T47D cells mounted a proportional BubR1-dependent checkpoint response; MDA-MB-231 cells showed reduced checkpoint signaling, consistent with constitutive spindle assembly checkpoint (SAC) attenuation in triple-negative breast cancer. Beyond mitosis, HURP depletion impaired collective migration and converted MDA-MB-231 cells from super-diffusive, amoeboid-like motility to sub-diffusive behavior, while minimally affecting the less aggressive cell lines. HURP-depleted MDA-MB-231 spheroids were significantly larger, less compact, and less spherical than controls, linking spindle regulation to tissue-level architectural coherence. These findings establish HURP as a multifunctional regulator coordinating mitotic fidelity, migration plasticity, and tumor architecture in breast cancer, with a selective dependency in highly metastatic cells, positioning it as a promising therapeutic target for aggressive breast cancers.

## 1. Introduction

Breast cancer (BC) remains the most frequently diagnosed cancer and the leading cause of cancer-related mortality in women, according to the Global Cancer Statistics 2022 report [1]. Despite advances in early detection and treatment, the prognosis for patients with metastatic disease—particularly triple-negative breast cancer (TNBC)—remains poor, underscoring the need to identify the molecular mechanisms that drive tumor dissemination and define new therapeutic targets [2].

A hallmark of cancer progression is chromosomal instability (CIN), characterized by an increased rate of chromosome missegregation, often arising from mitotic errors, including multipolar spindle formation, impaired chromosome congression, defective kinetochore-microtubule attachments, and compromised mitotic checkpoints [3]. These mitotic aberrations are not merely passive consequences of malignancy—they actively generate the genomic heterogeneity that fuels clonal evolution, metastasis, and therapy resistance. Central to mitotic fidelity is the mitotic spindle, a dynamic microtubule-based structure whose precise assembly and orientation depend on a network of microtubule-associated proteins (MAPs) and regulatory kinases [4,5,6].

Among these, Hepatoma Upregulated Protein (HURP, also known as DLGAP5) has emerged as a critical player in spindle formation, chromosome congression, and mitotic progression [7,8,9]. HURP is frequently overexpressed in aggressive cancers, including breast cancer, and its elevated levels have been associated with increased proliferation, invasion, and poor prognosis [10,11,12,13,14,15,16,17]. Its recruitment to spindle microtubules is regulated by the Ran-GTP gradient and depends on Aurora-A-mediated phosphorylation at Ser627, linking HURP directly to the principal mitotic kinase axis [18].

HURP does not act alone but operates within a broader network of Ran-regulated spindle assembly factors (SAFs). Targeting Protein for Xenopus kinesin-like protein 2 (TPX2) is an essential microtubule nucleation and stabilization factor that activates the mitotic kinase Aurora-A [19,20,21,22]. Recent structural and functional studies demonstrate that TPX2 forms condensates on microtubules that recruit HURP to promote branching microtubule nucleation, identifying a close physical and functional relationship between these two factors [23]. Aurora-A kinase, stabilized by TPX2 at the spindle, is required for bipolar spindle formation, centrosome maturation, and chromosome segregation; its overexpression in breast cancer promotes tumor progression and is associated with enhanced cell migration [24]. We have previously established that HURP forms a Ran-GTP-dependent complex with TPX2 and Aurora-A in mammalian cells during mitosis [18], positioning HURP as a structural and regulatory component of this oncogenic signaling unit.

Nuclear Mitotic Apparatus protein (NuMA) completes this regulatory network by focusing spindle poles and mediating astral microtubule–cortex interactions through recruitment of the dynein–dynactin motor complex [25,26,27,28,29]. Aurora-A phosphorylates NuMA to regulate spindle orientation relative to the substrate, ensuring proper positioning of daughter cells after division [25,30]. Correct spindle orientation is therefore essential for epithelial tissue organization: errors in spindle positioning generate daughter cell displacement, promote tissue disorganization, and synergize with oncogenic mutations to initiate invasive behaviors [31,32].

Despite this mechanistic framework, the cell-type-specific contributions of HURP to spindle regulation, checkpoint fidelity, and metastatic behavior across the spectrum of breast cancer malignancy remain poorly defined. In particular, how HURP overexpression is reconciled with spindle dysfunction in highly metastatic cells, and whether HURP plays roles in cell migration and three-dimensional tumor organization beyond its established mitotic functions, have not been systematically investigated.

In this study, we employ a breast cell line model spanning a metastatic gradient—non-transformed MCF10A, low-metastatic luminal A T47D, and highly metastatic triple-negative MDA-MB-231 cells—to systematically dissect the role of HURP and its functional interplay with TPX2, Aurora-A, and NuMA in mitotic spindle organization, cell-cycle regulation, spindle checkpoint function, migration dynamics, and three-dimensional tumor architecture. Our results reveal HURP as a multifunctional regulator whose disruption has profoundly different consequences depending on the metastatic state of the cell line, establishing a rationale for selectively targeting HURP in aggressive breast cancer.

## 2. Results

### 2.1. HURP Expression and Localization Correlate with Metastatic Potential

To investigate HURP’s association with breast cancer progression, we analyzed its expression and spindle localization in three breast cell lines with increasing metastatic potential: MCF10A, T47D, and MDA-MB-231. MCF10A is an immortalized, non-transformed human breast epithelial cell line that resembles normal breast epithelium and serves as a control in in vitro breast cancer studies [33,34]. T47D is a low-metastatic breast cancer cell line, classified as luminal A based on the presence of estrogen and progesterone receptors and absence of human epithelial receptor 2 (ER+, PR+, HER2−) [34,35]. MDA-MB-231 is a highly aggressive, triple-negative breast cancer cell line (ER-, PR-, and HER2−), representing a model for advanced, metastatic disease [34,36].

**Figure 1:**
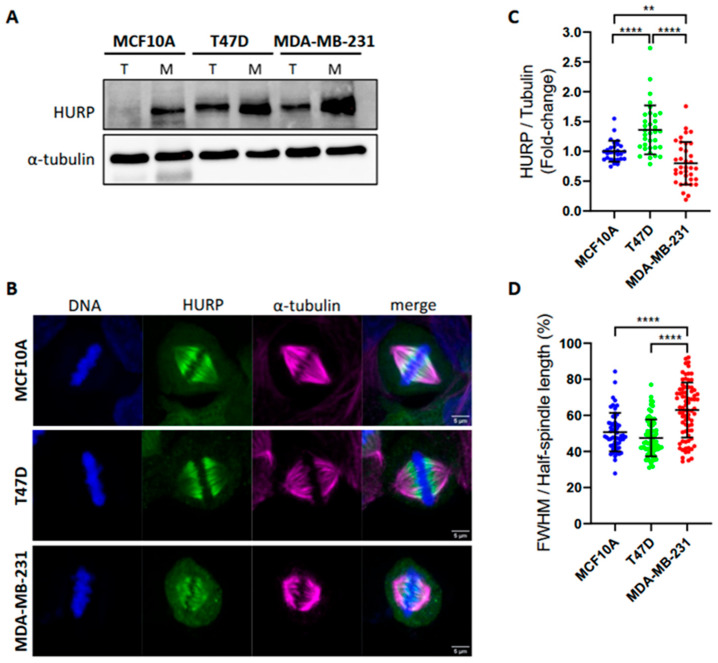
HURP expression, spindle association, and axial distribution correlate with metastatic potential in breast cell lines. (**A**) Immunoblot analysis of total (T) and mitotic (M) protein extracts from MCF10A, T47D, and MDA-MB-231 cells, probed for HURP. α-tubulin serves as a loading control. (**B**) Representative immunofluorescence images of metaphase-arrested MCF10A, T47D, and MDA-MB-231 cells stained for HURP (green), α-tubulin (magenta), and DNA (blue, Hoechst). HURP localizes to kinetochore fiber plus-ends in all cell lines. Scale bar, 5 μm. (**C**) Scatter plots of spindle-bound HURP fluorescence intensity, normalized to α-tubulin in metaphase cells (MCF10A, *n* = 28; T47D, *n* = 34; MDA-MB-231, *n* = 36). Data are presented as mean intensities ± SD. Statistical significance determined by two-tailed Mann–Whitney test: ** 0.001 < *p* < 0.01; **** *p* < 0.0001. (**D**) Scatter plots of HURP spatial distribution along the metaphase spindle axis, expressed as the ratio of HURP full width at half maximum (FWHM) to half-spindle length (MCF10A, *n* = 28; T47D, *n* = 34; MDA-MB-231, *n* = 39). A higher ratio indicates broader HURP distribution relative to spindle length. Data are presented as mean intensities ± SD. **** *p* < 0.0001, two-tailed Mann–Whitney test.

Western blot analysis of both total and mitotic protein extracts revealed significantly higher HURP levels in cancer cell lines compared to non-cancerous MCF10A, with MDA-MB-231 showing the highest expression (Figure 1A). Immunofluorescence confirmed HURP localization at the plus ends of k-fibers in all three cell lines (Figure 1B). Interestingly, despite higher mitotic HURP protein levels, spindle-bound HURP fluorescence intensity was reduced by 20% in MDA-MB-231 cells relative to MCF10A, suggesting cytoplasmic mislocalization, whereas T47D cells showed a 40% increase in spindle-bound HURP (Figure 1C). Spindle tubulin levels were lowest in T47D cells, intermediate in MDA-MB-231 cells, and highest in MCF10A cells (Figure S1A).

Spatial distribution analysis of HURP along the spindle axis revealed that HURP occupied 63% of the half-spindle length in MDA-MB-231 cells, compared to 51% and 47% in MCF10A cells and T47D cells, respectively (Figure 1D and Figure S1B). These data show that MDA-MB-231 cells exhibit the lowest spindle-bound HURP per tubulin unit and show a broader redistribution of HURP along the metaphase spindle axis, despite elevated total protein levels.

### 2.2. HURP Depletion Induces Cell-Line-Specific Spindle Defects

To assess HURP’s functional contribution to spindle integrity, we analyzed the effect of siRNA-mediated HURP silencing across our cell line panel.

**Figure 2:**
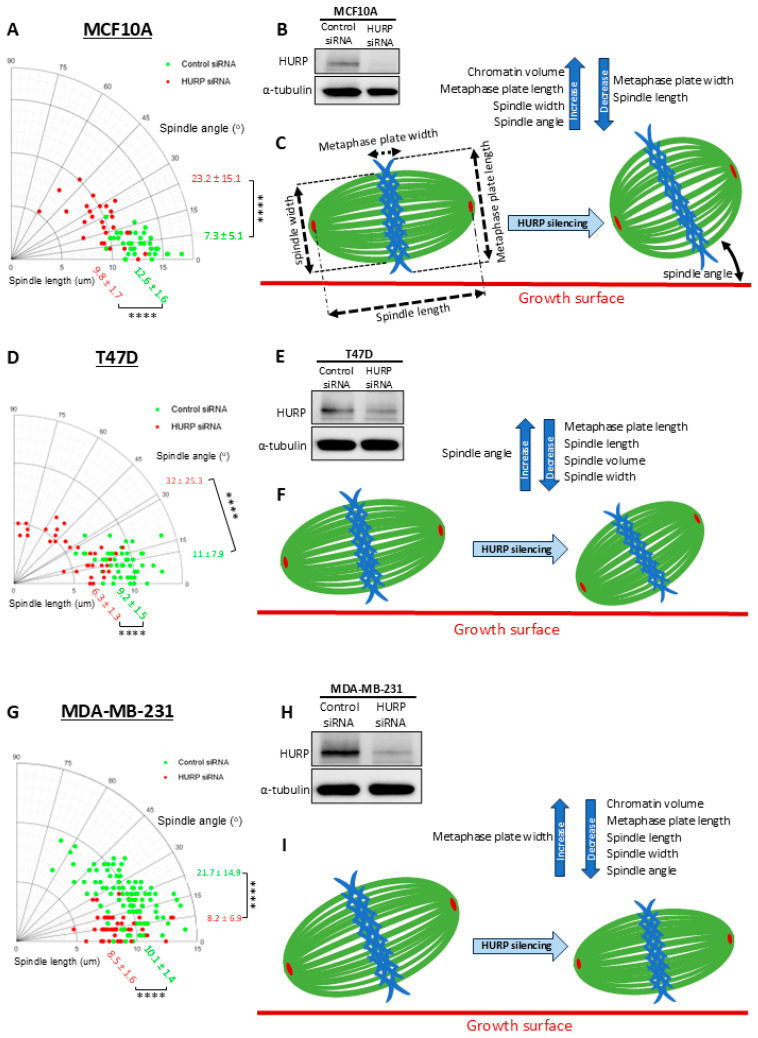
HURP depletion induces cell line-specific spindle misorientation and architectural defects. (**A**,**D**,**G**) Polar scatter plots of 3D-measured spindle angle (degrees from growth surface) versus spindle length (μm) for control (green) or HURP siRNA (red) conditions in (**A**) MCF10A (control, *n* = 41; HURP siRNA, *n* = 27), (**C**) T47D (control, *n* = 51; HURP siRNA, *n* = 37), and (**E**) MDA-MB-231 (control, *n* = 107; HURP siRNA, *n* = 50). Filled points indicate single cell values ± SD. **** *p* < 0.0001, two-tailed Mann-Whitney test. (**B**,**E**,**H**) Immunoblot analysis of total protein extracts from control and HURP siRNA-treated MCF10A, T47D and MDA-MB-231 cells, probed for HURP with α-tubulin as loading control. (**C**,**F**,**I**) Schematic representation of quantitative 3D spindle architecture parameters—chromatin volume, metaphase plate width and length, mean spindle width, and spindle volume—measured using the Spindle3D plugin (Kletter et al., 2022 [37]) in bipolar spindles from MCF10A (**B**), T47D (**D**), and MDA-MB-231 (**F**) cells treated with control or HURP siRNA.

In MCF10A cells, HURP silencing (confirmed by Western blot, Figure 2B) increased the percentage of abnormal spindles by 27.9% compared to control conditions, with multipolar configurations as the predominant aberrant phenotype (Figure S2A,B). Spindle length decreased significantly from 12.6 μm (control siRNA) to 9.8 μm (HURP siRNA; *p* < 0.0001; Figure 2A), and spindle angle increased from 7.3° to 23.2° (*p* < 0.0001; Figure 2A, Sup. movies 1–2). Three-dimensional spindle architecture analysis [37] revealed that HURP silencing expanded chromatin volume, increased metaphase plate length and spindle width, and decreased metaphase plate width (Figure 2C and Figure S2C).

In low-metastatic T47D cells, HURP silencing was confirmed by both immunofluorescence and Western blot (Figure S3A,E). HURP silencing exacerbated inherent spindle defects, increasing the proportion of abnormal spindles from 50% to 66% (Figure S3A,B). Spindle length decreased by 31.5% (from 9.2 μm to 6.3 µm; *p* < 0.0001; Figure 2D), and spindle angle increased from 11° to 32° (*p* < 0.0001; Figure 2D, Sup. movies 3–4). Three-dimensional spindle architecture analysis revealed reduced metaphase plate length, spindle volume, and spindle width (Figure 2F and Figure S3C).

In highly metastatic MDA-MB-231 cells, HURP silencing was confirmed by immunofluorescence and Western blot (Figure S4A,H). Control MDA-MB-231 cells already exhibited predominantly abnormal, multipolar spindles (83.4%; Figure S4A,B, control siRNA). Following HURP silencing, apoptosis increased from 2.6% to 36.4% of the cell population (Figure S4A,B, HURP siRNA). In surviving cells, spindle angle decreased from 21.7° to 8.2° (*p* < 0.0001; Figure 2G) and spindle length shortened from 10.1 μm to 8.5 µm (*p* < 0.0001; Figure 2G, Sup. movies 5–6). These flattened spindles exhibited significantly reduced chromatin volume, metaphase plate length, and spindle width, while metaphase plate width increased (Figure 2I and Figure S4C).

To determine whether the spindle defects were accompanied by changes in cell-cycle distribution, flow cytometry was performed. No significant differences in cell-cycle phase distribution were detected between control and HURP-silenced MCF10A cells (Figure S5A,B). In contrast, MDA-MB-231 cells showed a marked reduction in total cell counts following HURP silencing, consistent with increased cell death (Figure S5C). Among surviving cells, the G1 population was reduced (62% → 11.3%; *p* < 0.01), while S-phase (15.5% → 31%; *p* < 0.05) and G2/M phase populations increased (22.5% → 57.7%; *p* < 0.01), indicating G2/M arrest (Figure S5C,D).

### 2.3. HURP Depletion Alters Spindle Checkpoint Activation

To understand the mechanism underlying the increased mitotic spindle defects, we measured interkinetochore distances between sister chromatids and quantified BubR1 signal intensity at paired centromeres as readouts of kinetochore tension and spindle checkpoint status, respectively (Figure S5E).

**Figure 3:**
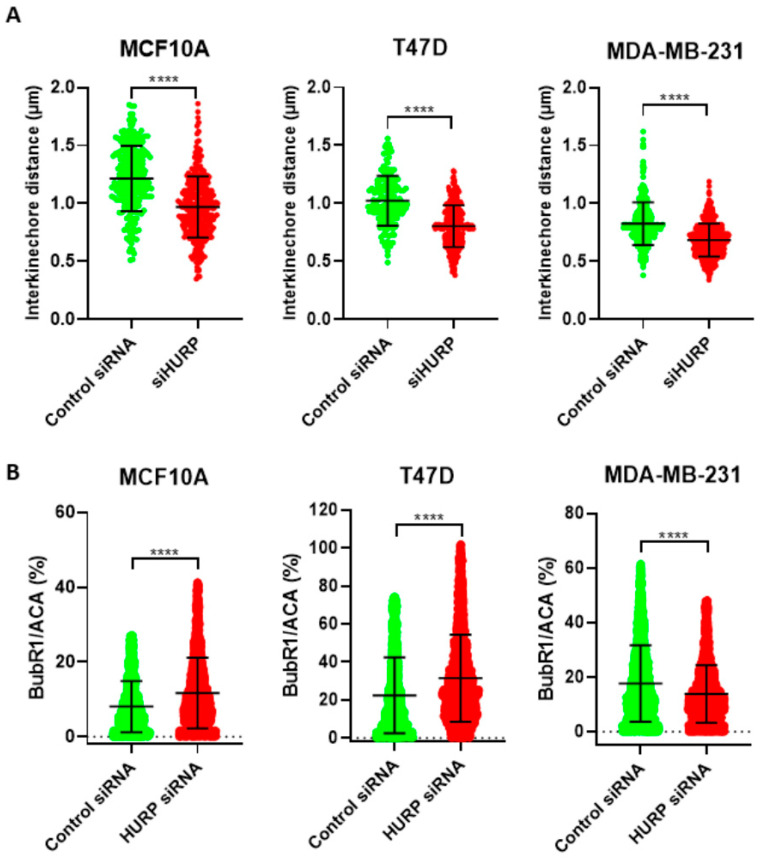
HURP depletion reduces interkinetochore tension in all cell lines and impairs spindle checkpoint activation selectively in highly metastatic MDA-MB-231 cells. (**A**) Scatter plot of 3D interkinetochore distance (μm) between paired sister kinetochores in control and HURP siRNA-treated MCF10A, T47D, and MDA-MB-231 cells. (MCF10A: control, *n* = 23 cells; HURP siRNA, *n* = 34 cells; T47D: control, *n* = 26 cells; HURP siRNA, *n* = 32 cells; MDA-MB-231: control, *n* = 38 cells; HURP siRNA, *n* = 45 cells). Kinetochores were detected using ACA immunostaining and linked in 3D using a custom linear assignment algorithm. Data are presented as mean ± SD. ****: *p* < 0.0001, two-tailed Mann–Whitney test. (**B**) Scatter plots of kinetochore-bound BubR1 signal against ACA signal in control and HURP siRNA-treated cells from each cell line (same *n* as in (**A**)). Data are presented as mean intensities ± SD. ****: *p* < 0.0001, two-tailed Mann–Whitney test. Note the divergent response between non-transformed/low-metastatic cells (BubR1 increases) and highly metastatic MDA-MB-231 cells (BubR1 decreases) upon HURP depletion.

HURP silencing significantly reduced interkinetochore distances in all three cell lines (MCF10A: from 1.2 μm to 0.97 μm; T47D: from 1.0 μm to 0.8 μm; MDA-MB-231: from 0.83 μm to 0.69 μm; all *p* < 0.0001; Figure 3A), indicating decreased tension between sister kinetochores across all conditions.

Kinetochore-bound BubR1 quantification analysis displayed opposing responses. In non-transformed MCF10A and low-metastatic T47D cells, HURP silencing led to a significant increase in BubR1 signal (MCF10A: from 8% to 11.7%; T47D: from 22.4% to 31.5%; *p* < 0.0001; Figure 3B), consistent with spindle assembly checkpoint (SAC) activation in response to reduced kinetochore tension. In MDA-MB-231 cells, BubR1 signal decreased significantly following HURP depletion (from 17.6% to 13.8%; *p* < 0.0001; Figure 3B), despite the concomitant reduction in interkinetochore distance, indicating a failure to mount a proportional checkpoint response.

### 2.4. HURP Modulates Spindle-Associated Protein Dynamics

To investigate how HURP regulates spindle organization at the molecular level, we examined the spindle-bound levels and localization of TPX2, Aurora-A, and NuMA—proteins that form a functional complex with HURP during mitosis [18] and cooperate to regulate spindle assembly and orientation [20,25,30].

**Figure 4:**
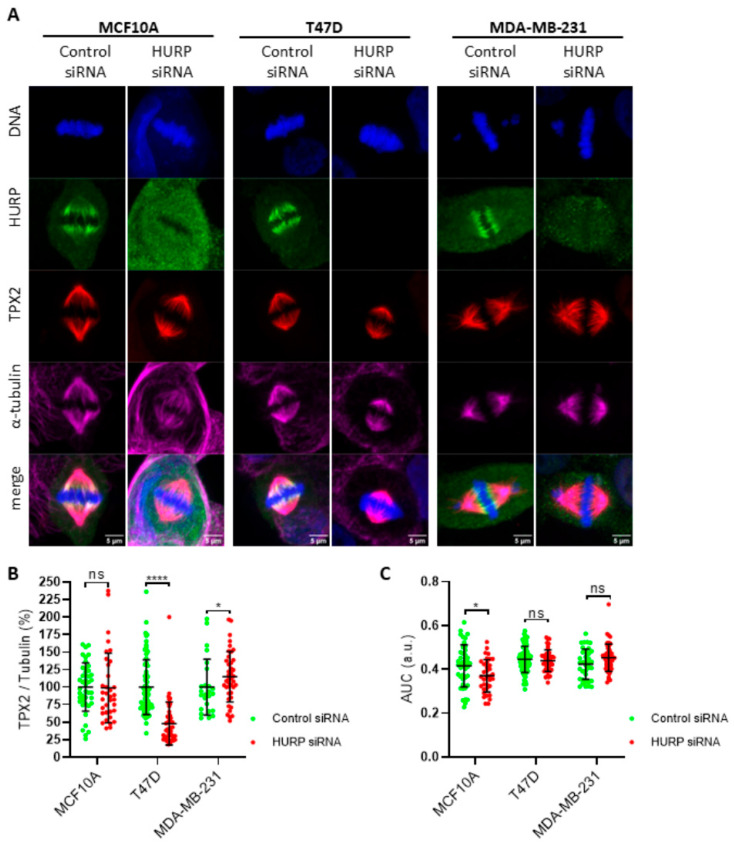
HURP silencing differentially alters spindle-bound TPX2 levels and distribution across breast cell lines. (**A**) Representative immunofluorescence images of metaphase-arrested MCF10A, T47D, and MDA-MB-231 cells treated with control or HURP siRNA, stained for HURP (green), TPX2 (red), α-tubulin (magenta), and DNA (blue, Hoechst). Scale bar, 5 μm. (**B**) Scatter plots of spindle-bound TPX2 fluorescence intensity, normalized to α-tubulin (a.u.), in each cell line and condition (MCF10A: control, *n* = 43; HURP siRNA, *n* = 35; T47D: control, *n* = 73; HURP siRNA, *n* = 45; MDA-MB-231: control, *n* = 25; HURP siRNA, *n* = 41). Data are presented as mean intensities ± SD. * 0.01 < *p* < 0.05; **** *p* < 0.0001; ns, not significant; two-tailed Mann–Whitney test. (**C**) Scatter plots of TPX2 axial distribution along the metaphase spindle, quantified as the area under the curve (AUC) of normalized pole-to-pole intensity profiles of each spindle (MCF10A: control, *n* = 43; HURP siRNA, *n* = 35; T47D: control, *n* = 76; HURP siRNA, *n* = 45; MDA-MB-231: control, *n* = 36; HURP siRNA, *n* = 51). Data are presented as mean ± SD. * 0.01 < *p* < 0.05; ns, not significant; Mann–Whitney test, two tailed.

In MCF10A cells, HURP silencing did not significantly alter total spindle-bound TPX2 levels but induced a subtle redistribution toward the spindle poles (*p* < 0.05; Figure 4A–C and Figure S6A). In T47D cells, HURP depletion reduced spindle-bound TPX2 by 52% (*p* < 0.0001; Figure 4B), while pole-localized distribution was maintained (Figure 4A,C and Figure S6A). In MDA-MB-231 cells, spindle-bound TPX2 increased by 14.7% following HURP silencing (*p* < 0.05; Figure 4B), with preserved pole-localization patterns (Figure 4A,C and Figure S6A).

**Figure 5:**
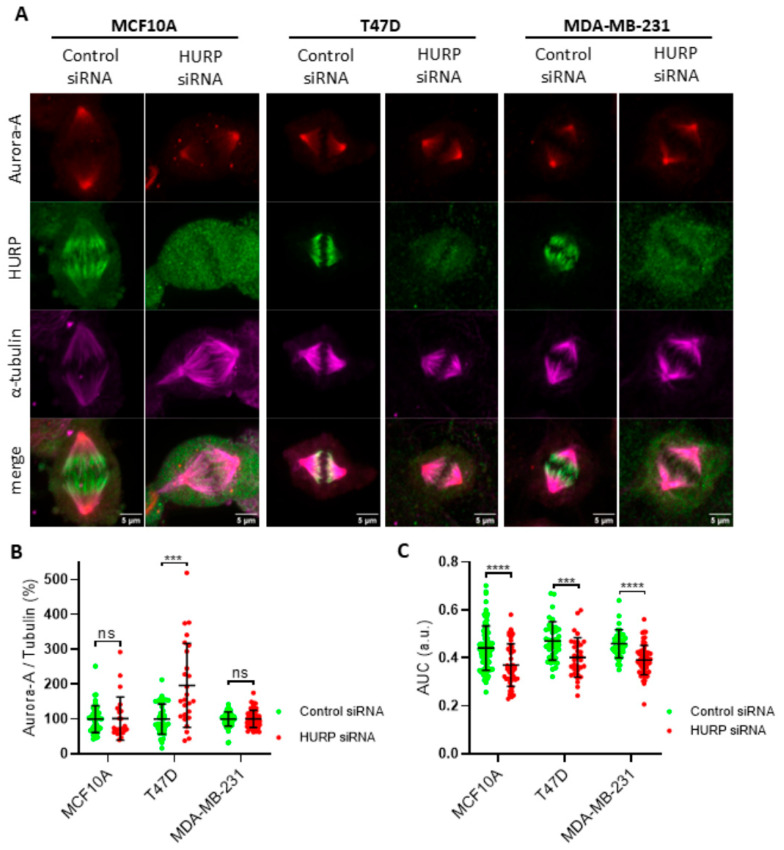
Aurora-A is restricted to spindle poles and is elevated in T47D cells following HURP silencing. (**A**) Representative immunofluorescence images of metaphase-arrested MCF10A, T47D, and MDA-MB-231 cells treated with control or HURP siRNA, stained for HURP (green), Aurora-A (red), and α-tubulin (magenta). Scale bar, 5 μm. (**B**) Scatter plots of spindle-bound Aurora-A fluorescence intensity, normalized to α-tubulin (a.u.), in each cell line and condition (MCF10A: control, *n* = 50; HURP siRNA, *n* = 27; T47D: control, *n* = 38; HURP siRNA, *n* = 26; MDA-MB-231: control, *n* = 58; HURP siRNA, *n* = 62). Data are presented as mean intensities ± SD. *** 0.0001 < *p* < 0.001; ns, not significant; two-tailed Mann–Whitney test. (**C**) Scatter plots of Aurora-A axial distribution quantified as the AUC of normalized pole-to-pole intensity profiles (MCF10A: control, *n* = 84; HURP siRNA, *n* = 49; T47D: control, *n* = 38; HURP siRNA, *n* = 33; MDA-MB-231: control, *n* = 34; HURP siRNA, *n* = 62). Data are presented as mean ± SD. *** 0.0001 < *p* < 0.001; **** *p* < 0.0001, two-tailed Mann–Whitney test.

Analysis of Aurora-A revealed that HURP depletion narrowed its spindle distribution to a more concentrated region at the poles in all three cell lines (Figure 5A,C and Figure S6B). In T47D cells, spindle-bound Aurora-A levels increased by 96% (*p* < 0.001; Figure 5B), an increase occurring in the context of reduced TPX2 levels. Aurora-A levels were not significantly altered in MCF10A or MDA-MB-231 cells following HURP silencing (Figure 5B).

**Figure 6:**
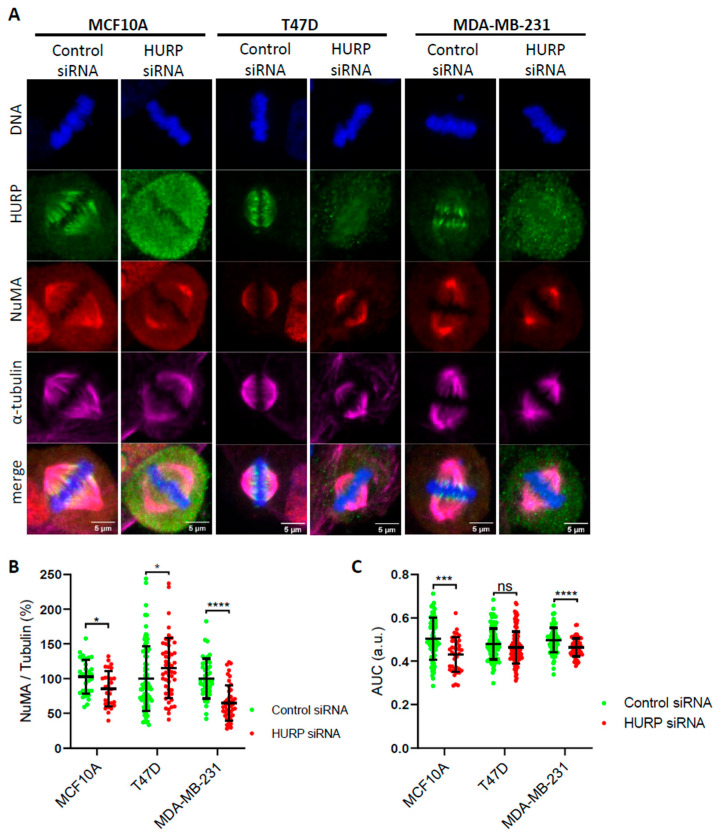
NuMA spindle levels and pole-region distribution are selectively reduced in MCF10A and MDA-MB-231 cells following HURP depletion. (**A**) Representative immunofluorescence images of metaphase MCF10A, T47D, and MDA-MB-231 cells treated with control or HURP siRNA, stained for HURP (green), NuMA (red), α-tubulin (magenta), and DNA (blue, Hoechst). Scale bar, 5 μm. (**B**) Scatter plots of spindle-bound NuMA fluorescence intensity normalized to α-tubulin (a.u.), in each cell line and condition (MCF10A: control, *n* = 32; HURP siRNA, *n* = 28; T47D: control, *n* = 82; HURP siRNA, *n* = 51; MDA-MB-231: control, *n* = 41; HURP siRNA, *n* = 45). Data are presented as mean intensities ± SD. * 0.01 < *p* < 0.05; **** *p* < 0.0001, two-tailed Mann–Whitney test. (**C**) Scatter plots of NuMA axial distribution quantified as the AUC of normalized pole-to-pole intensity profiles (MCF10A: control, *n* = 68; HURP siRNA, *n* = 37; T47D: control, *n* = 98; HURP siRNA, *n* = 124; MDA-MB-231: control, *n* = 92; HURP siRNA, *n* = 65). Data are presented as mean ± SD. *** 0.0001 < *p* < 0.001; **** *p* < 0.0001; ns, not significant; two-tailed Mann–Whitney test.

HURP silencing reduced spindle-bound NuMA levels by 14% in MCF10A (*p* < 0.05) and by 35% in MDA-MB-231 cells (*p* < 0.0001) while increasing NuMA by 13% in T47D cells (*p* < 0.05; Figure 6A,B). After HURP silencing, T47D cells retained a narrow NuMA distribution at the spindle poles, whereas MCF10A and MDA-MB-231 cells lost the broad NuMA distribution in the pole region, with the protein becoming strictly pole-confined (Figure 6A,C and Figure S6C). Spindle tubulin levels remained unchanged across all conditions and cell lines (Figure S6D), confirming that alterations in spindle-associated protein levels were not secondary to changes in microtubule content.

### 2.5. HURP Silencing Impairs Collective and Individual Cell Migration

To understand the role of HURP in breast cancer progression beyond mitosis, we performed scratch wound-healing assays in the presence or absence of mitomycin-C to distinguish migration from proliferation effects.

**Figure 7:**
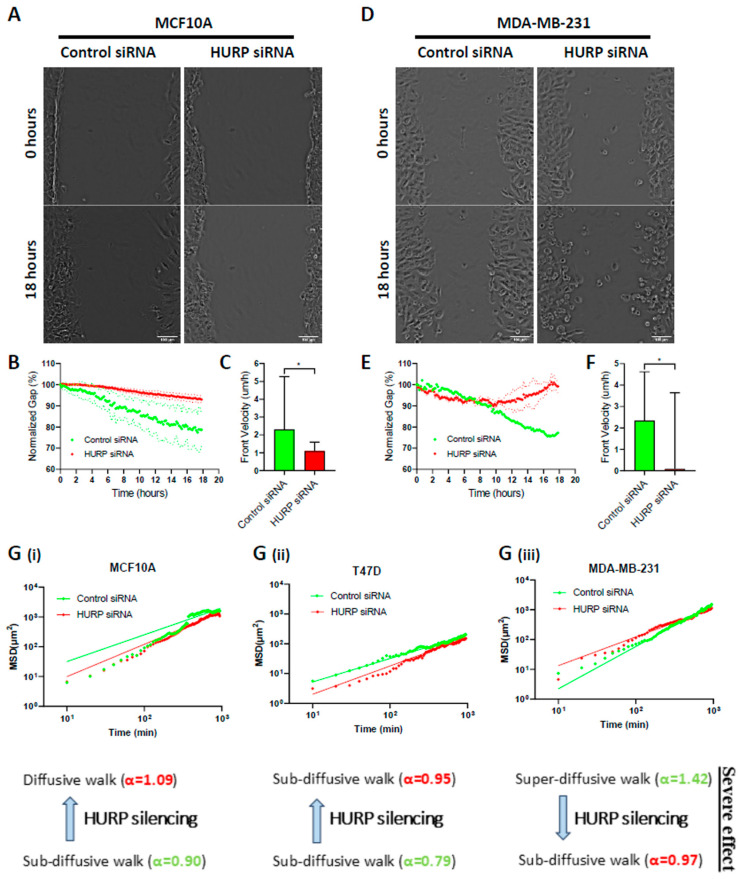
HURP silencing impairs collective migration and alters single-cell motility in breast cell lines. (**A**,**D**) Representative phase-contrast time-lapse images from scratch wound-healing assays of mitomycin-C-treated MCF10A (**A**) and MDA-MB-231 (**D**) cells transfected with control or HURP siRNA, at 0 and 18 h. Scale bar, 100 μm. (**B**,**E**) Quantification of gap closure (%) over time for MCF10A (**B**) and MDA-MB-231 (**E**) cells (MCF10A: control, *N* = 3 fields; HURP siRNA, *N* = 2; MDA-MB-231: control, *N* = 1; HURP siRNA, *N* = 2). (**C**,**F**) Mean front velocity (μm/h) of MCF10A (**C**) and MDA-MB-231 (**F**) cells after control or HURP siRNA treatment (MCF10A: control, *n* = 52; HURP siRNA, *n* = 35; MDA-MB-231: control, *n* = 18; HURP siRNA, *n* = 36). * 0.01 < *p* < 0.05, two-tailed Mann–Whitney test. Error bars denote SD. (**G**) Log-log plots of mean square displacement (MSD; μm^2^) versus time interval (min) for single-cell tracking experiments in mitomycin-C-treated cells, with fitted power-law exponents (α) and 95% confidence intervals: (**i**) MCF10A (control siRNA, α = 0.90, 95% CI 0.70 to 1.13; HURP siRNA, α = 1.09, 95% CI 0.99 to 1.18); (**ii**) T47D (control siRNA, α = 0.79, 95% CI 0.72 to 0.85; HURP siRNA, α = 0.95, 95% CI 0.87 to 1.03); (**iii**) MDA-MB-231 (control siRNA, α = 1.42, 95% CI 1.30 to 1.54; HURP siRNA, α = 0.97, 95% CI 0.90 to 1.02). Values of α < 1, α = 1, and α > 1 correspond to sub-diffusive, diffusive, and super-diffusive motion, respectively. See Sup. Movie 19.

Under mitomycin-C treatment, HURP silencing reduced gap closure in MCF10A by 67% (from 21% to 7%; Figure 7A,B) and decreased front velocity from 2.3 μm/h to 1.1 μm/h (*p* < 0.05; Figure 7A,C, Sup. movies 7–8). In MDA-MB-231 cells, HURP silencing severely impaired collective migration, with cells failing to close the scratch gap and displaying a marked reduction in front velocity (Figure 7D–F, Sup. Movies 9–10). It should be noted that HURP silencing in MDA-MB-231 cells is associated with a significant increase in apoptosis (Figure S5C,D), which will reduce the number of viable cells contributing to gap closure. The observed impairment in collective migration therefore likely reflects a combination of reduced cell viability and an intrinsic migratory deficit in surviving HURP-depleted cells. In T47D cells, HURP silencing did not significantly affect gap closure or front velocity under mitomycin-C conditions (front velocity: control siRNA, not significant; Figure S7A–C, Sup. Movies 15–18).

In the absence of mitomycin-C, both MCF10A and MDA-MB-231 cells exhibited higher gap closure rates and velocities, regardless of HURP status, reflecting contributions from cell proliferation (MCF10A: control siRNA 31%, HURP siRNA 23.8%, front velocities 4.4 μm/h and 3.9 μm/h, respectively; MDA-MB-231: control siRNA 57.3%, HURP siRNA 52.5%, velocities 8 μm/h and 6.3 μm/h, respectively; *p* < 0.05; Figure S8A–F, Sup. Movies 11–14). Differences between control and HURP-silenced conditions were less pronounced under these conditions, highlighting that HURP’s contribution to migration is more apparent when migration is uncoupled from proliferation. T47D cells showed no significant difference in migration parameters in the absence of mitomycin-C (Figure S7D–F, Sup. movies 15–18).

To characterize individual cell motility independently of apoptosis-related cell loss, single-cell tracking and mean square displacement (MSD) analysis were performed on viable, motile cells (Sup. Movie 19). Tracks were included only when they exceeded a minimum duration of 940 min; apoptotic cells, which characteristically round up and detach from the substrate prior to this threshold, were excluded from the analysis by design. This criterion ensures that the MSD exponents reported below reflect the intrinsic motility of surviving cells. In MCF10A cells, HURP silencing shifted cell motility toward more diffusive behavior (control: α = 0.90, 95% CI: 0.70–1.13; HURP siRNA α = 1.09, 95% CI: 0.99–1.18; Figure 7G(i)). T47D cells maintained sub-diffusive migration under both conditions (control siRNA: α = 0.79, 95% CI: 0.72–0.85; HURP siRNA: α = 0.95, 95% CI: 0.87–1.03; Figure 7G(ii)). Strikingly, in MDA-MB-231 cells, HURP silencing converted the migration profile from super-diffusive (α = 1.42, 95% CI: 1.30–1.54), typical of highly invasive cells, to sub-diffusive behavior (α = 0.97, 95% CI: 0.90–1.02; Figure 7G(iii)). This conversion demonstrates that viable HURP-depleted MDA-MB-231 cells exhibit a genuine intrinsic defect in directional motility, distinct from and in addition to any effect attributable to increased apoptosis.

### 2.6. HURP Silencing Disrupts Compact Multicellular Spheroid Formation

**Figure 8:**
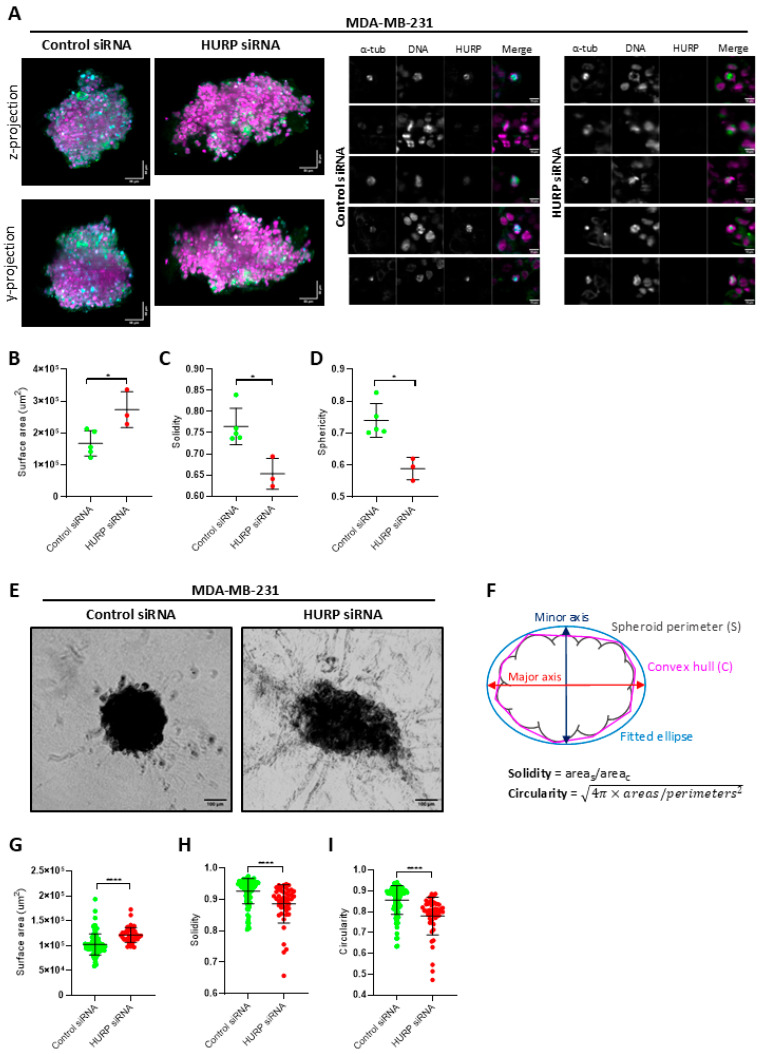
HURP depletion disrupts compact multicellular spheroid formation in MDA-MB-231 cells. (**A**) Maximum-intensity projection images (*z*- and *y*-axis views) of MDA-MB-231 spheroids pre-treated with control siRNA or HURP siRNA and imaged by single-plane illumination microscopy (left panel). Montages of zoomed mitotic spindles from the spheroid surface (right panel). HURP (cyan), α-tubulin (green), DNA (SPY555, magenta). Scale bars, 50 μm (overview), 10 μm (zoomed mitotic spindles). (**B**–**D**) Scatter plots of 3D spheroid geometrical parameters in control (*n* = 5) and HURP siRNA (*n* = 3) conditions: surface area (μm^2^) (**B**), solidity (**C**), and sphericity (**D**). Data are presented as mean ± SD. * 0.01 < *p* < 0.05, two-tailed Mann–Whitney test. (**E**) Representative phase-contrast images of MDA-MB-231 spheroids cultured for 48 h in 2% Matrigel, pre-treated with control (left) or HURP siRNA (right). (**F**) Schematic of 2D geometric quantification metrics. The subscripts (S) and (C) denote spheroid and convex hull measurements, respectively. (**G**–**I**) Scatter plots of 2D spheroid geometrical parameters in control (*n* = 92) and HURP siRNA (*n* = 47) spheroids: projected area (μm^2^) (**G**), solidity (**H**), and circularity (**I**). Data are presented as mean ± SD. ****: *p* < 0.0001, two-tailed Mann–Whitney test.

To examine HURP’s role in three-dimensional tissue organization, MDA-MB-231 spheroid formation was analyzed following HURP depletion. Single-plane illumination microscopy confirmed the absence of HURP from mitotic spindles after siRNA treatment (Figure 8A). Three-dimensional geometrical analysis revealed that HURP-depleted spheroids were significantly larger (surface area increased by 58.8%, from 1.7 × 10^5^ to 2.7 × 10^5^ µm^2^; *p* < 0.05; Figure 8B), but less compact (solidity: from 0.76 to 0.65; *p* < 0.05; Figure 8C) and less spherical (sphericity: from 0.74 to 0.59; *p* < 0.05; Figure 8D) than controls (Sup. Movies 20–23).

Two-dimensional morphological quantification from phase-contrast images (Figure 8E,F) corroborated these findings: HURP-silenced spheroids were significantly larger (from 1 ×10^5^ μm^2^ to 1.2 × 10^5^ μm^2^; *p* < 0.0001), less solid (from 0.93 to 0.89; *p* < 0.0001), and less circular (from 0.86 to 0.78; *p* < 0.0001) than controls (Figure 8G–I).

**Figure 9:**
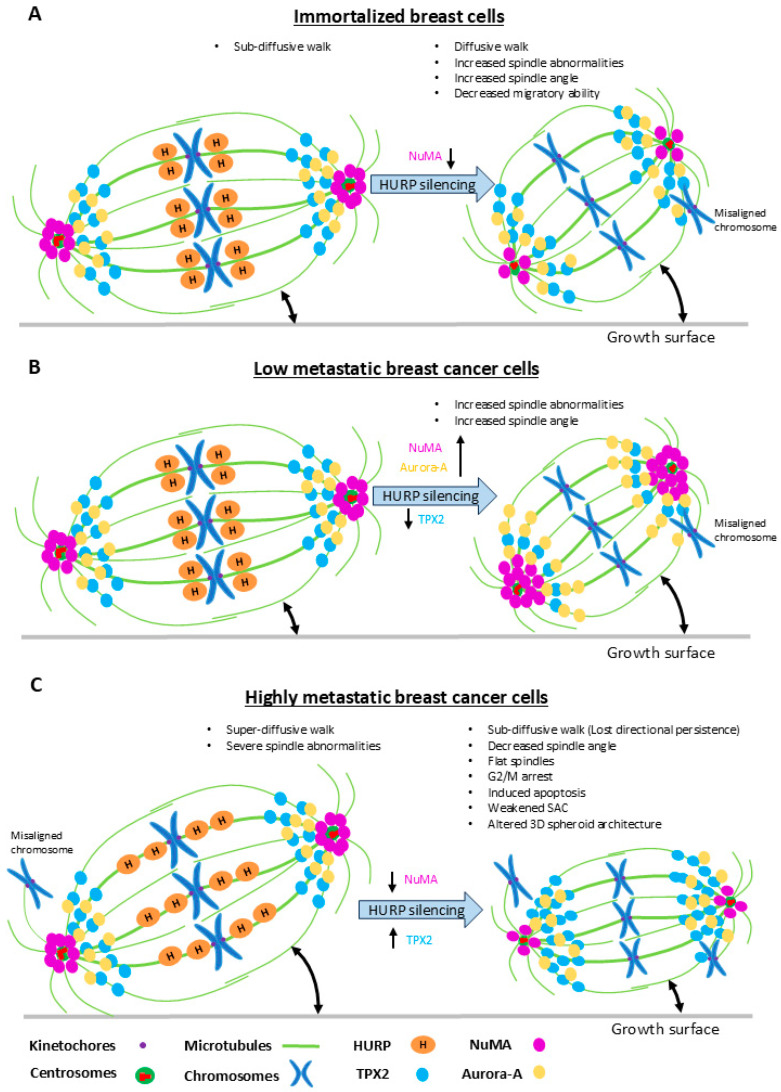
Schematic model of HURP function across a metastatic gradient. Schematic summarizing the cell-line-specific consequences of HURP depletion in MCF10A (**A**), T47D (**B**), and MDA-MB-231 (**C**) cells. For each cell line, the model depicts the baseline state and the effects of HURP silencing on mitotic spindle organization and angle; spindle-associated levels and distributions of TPX2, Aurora-A, and NuMA; BubR1 checkpoint response; cell-cycle distribution; individual migration mode (MSD α-value); and 3D spheroid architecture. Severity of mitotic and migratory phenotypes scales in parallel with metastatic potential, establishing a differential therapeutic vulnerability in triple-negative breast cancer.

Taken together, the cell-line-specific effects of HURP silencing on spindle organization, checkpoint fidelity, migration dynamics, and tissue architecture are summarized in Figure 9.

## 3. Discussion

This study provides new insights into the role of HURP and its associated spindle protein network in breast cancer progression, demonstrating that aberrant HURP regulation contributes to aggressive tumor phenotypes across mitotic, migratory, and tissue-architectural dimensions. Using a breast cell line model spanning a metastatic gradient, we show that HURP is not only overexpressed in highly metastatic cells, but its spindle association, regulation of downstream effectors, and control of migratory plasticity are fundamentally altered in aggressive breast cancer subtypes.

Consistent with previous reports showing elevated HURP/DLGAP5 mRNA and protein levels in breast tumors and aggressive cell lines [11,12], HURP expression increased progressively across the metastatic gradient, with MDA-MB-231 cells displaying the highest levels. This pattern is not unique to breast cancer: elevated HURP expression has been documented across multiple tumor types, including hepatocellular carcinoma, colorectal cancer, and non-small-cell lung cancer [10,15,16], suggesting a shared oncogenic selection pressure for HURP upregulation in aggressive malignancies. At the mechanistic level, this overexpression is primarily driven by transcriptional upregulation, as demonstrated by elevated DLGAP5 mRNA across cancer datasets [12]. Post-translational stabilization is likely to compound this effect: HURP undergoes APC/C–Cdh1-mediated proteasomal degradation during G1, and in cancer cells where CDK activity is elevated and APC/C–Cdh1 function is consequently dysregulated, HURP turnover may be impaired, further contributing to its accumulation [10].

Paradoxically, spindle-bound HURP levels were reduced in MDA-MB-231 cells despite elevated total protein, indicating a shift toward cytoplasmic mislocalization. This finding is mechanistically important: Aurora-A-dependent phosphorylation of HURP at Ser627 governs its spindle recruitment in a Ran-GTP-dependent manner [18,23]. In triple-negative breast cancer, where Aurora-A is frequently overexpressed, constitutive hyperphosphorylation of HURP may alter its affinity for spindle-associated microtubules, driving cytoplasmic redistribution [24]. Rather than reflecting simple mitotic insufficiency, this redistribution may represent an adaptation that enables highly metastatic cells to sustain a permissive level of mitotic infidelity.

### 3.1. Metastatic Cells Display Selective Dependence on HURP for Mitotic Survival

HURP silencing induced cell-line-specific spindle defects, with the most severe consequences observed in highly metastatic MDA-MB-231 cells. In non-transformed MCF10A cells, HURP loss caused moderate spindle disorganization and increased spindle misorientation without triggering significant cell death. T47D cells showed exacerbation of their already elevated baseline of spindle abnormalities and pronounced spindle shortening. In contrast, MDA-MB-231 cells exhibited massive apoptosis (36.4% vs. 2.6% in controls) and G2/M arrest upon HURP depletion. This differential vulnerability is consistent with the concept of non-oncogene addiction, wherein chromosomally unstable cancer cells become disproportionately reliant on specific mitotic regulators to manage replicative stress and genomic instability [3]. The relative tolerance of MCF10A cells to HURP loss defines a potential therapeutic window for selectively targeting HURP in aggressive breast cancer subtypes.

### 3.2. Divergent Spindle Checkpoint Responses Reflect SAC Attenuation in Tnbc

HURP depletion reduced interkinetochore distances across all three cell lines, confirming that HURP contributes to kinetochore tension regardless of metastatic status. However, the downstream checkpoint response diverged markedly between cell types. In MCF10A and T47D cells, reduced kinetochore tension was accompanied by increased BubR1 recruitment, consistent with canonical SAC activation. In MDA-MB-231 cells, BubR1 levels paradoxically decreased after HURP silencing despite diminished interkinetochore tension, indicating a failure to mount a proportional checkpoint response.

The changes in BubR1 levels observed upon HURP depletion most likely represent a consequence of altered k-fiber stability, rather than a direct role of HURP in BubR1 recruitment or expression. HURP’s primary function at the spindle is to stabilize k-fibers and promote the generation of interkinetochore tension. BubR1 is recruited to unattached or tension-deficient kinetochores through the KNL1–MELT–Bub1/BubR1 axis in a tension-sensitive manner [38]. In MCF10A and T47D cells, HURP loss reduces k-fiber stability and kinetochore tension, thereby providing the canonical signal for BubR1 enrichment at kinetochores. Whether HURP might additionally influence BubR1 stoichiometry at kinetochores through indirect remodeling of the outer kinetochore complex—for example, by altering the occupancy of other k-fiber-associated proteins—remains an open question that warrants future investigation.

This uncoupling of tension loss from BubR1 recruitment in MDA-MB-231 cells likely reflects the constitutively attenuated SAC capacity of triple-negative breast cancer. TNBC cell lines are known to harbor a weakened checkpoint associated with high baseline chromosomal instability, which renders them sensitive to spindle poisons but impairs their ability to mount a BubR1-mediated mitotic arrest in response to compounding spindle stress [39]. The BubR1 reduction following HURP depletion in MDA-MB-231 cells may thus represent SAC collapse under conditions of cumulative mitotic stress, mechanistically analogous to mitotic slippage—where progressive checkpoint weakening permits mitotic exit without faithful chromosome segregation—consistent with the concurrent G2/M accumulation and widespread apoptosis observed in this cell line [40,41].

### 3.3. Mechanistic Integration of HURP with Essential Spindle Regulators

Mechanistically, our results reveal that HURP is central to a regulatory axis involving TPX2, Aurora-A, and NuMA, which cooperate to drive spindle assembly, pole organization, and spindle orientation [25,30]. The cell-type-specific nature of the responses to HURP depletion within this module is a central finding of our study.

In T47D cells, HURP silencing reduced spindle-bound TPX2 while increasing Aurora-A levels at spindle poles. The Aurora-A/TPX2 interaction is required for Aurora-A activation and correct microtubule targeting [20,30]; when TPX2 is limiting, unconstrained Aurora-A activity at the pole may disrupt the balance of microtubule dynamics, contributing to the spindle shortening and misorientation observed in T47D cells. The concurrent 13% increase in NuMA in T47D represents a further compensatory rearrangement within the pole-organizing machinery. In MDA-MB-231 cells, HURP loss increased TPX2 while NuMA decreased by 35%—the largest reduction across all conditions—with both Aurora-A and NuMA losing their extended pole distributions and becoming strictly pole-confined. The increase in TPX2 in MDA-MB-231 cells may reflect an attempt to compensate for HURP loss by accumulating TPX2 condensates on spindle microtubules to sustain branching nucleation [23]; however, this compensation is evidently insufficient given the catastrophic spindle defects observed. Together, these data indicate that HURP acts as a contextual stabilizer of the Aurora-A/TPX2/NuMA axis, with its loss producing distinct molecular signatures depending on the baseline regulatory state of each cell type.

### 3.4. Spindle Orientation and Tissue Architectural Control

Proper spindle orientation is essential for accurate daughter cell positioning and maintenance of epithelial architecture, as misoriented divisions disrupt epithelial organization and promote genomic instability and metastatic dissemination [31]. HURP silencing significantly increased spindle angles in MCF10A and T47D cells, while producing flattened, angle-reduced spindles in MDA-MB-231 cells, pointing to distinct mechanisms of orientation disruption across cell types. The loss of extended NuMA distribution at spindle poles in MCF10A and MDA-MB-231 cells provides a mechanistic basis for this misorientation: NuMA recruits dynein to the cell cortex and focuses spindle poles, serving as a central effector of spindle positioning [27,29]. Reduced NuMA pole-region distribution is therefore expected to destabilize astral microtubule–cortex interactions and impair correct spindle placement.

These mitotic orientation defects manifested at the tissue scale in three-dimensional spheroid experiments. HURP-depleted MDA-MB-231 spheroids were significantly larger, less compact, and less spherical—a phenotype that is expected when misoriented divisions accumulate over multiple proliferative rounds, displacing daughter cells from their normal spatial relationships and disrupting cell–cell contacts. The reduced sphericity and solidity of HURP-depleted spheroids may also reflect altered cortical tension and intercellular adhesion downstream of NuMA mislocalization, with potential consequences for the invasive capacity of disseminating cells.

### 3.5. Migration Plasticity and the Mitosis–Motility Nexus

Beyond mitosis, HURP depletion markedly impaired migration, especially in MDA-MB-231 cells. Cancer cell invasion occurs through distinct migration modes: collective migration, where cell–cell contacts are maintained, and individual migration, which includes mesenchymal (protease-dependent) and amoeboid (protease-independent) subtypes [42,43]. Collective migration was impaired by HURP silencing in both MCF10A and MDA-MB-231 cells under mitomycin-C conditions, whereas T47D cells—which rely predominantly on cohesive collective migration—were unaffected.

Single-cell MSD analysis revealed that MDA-MB-231 cells under control conditions exhibit super-diffusive motility (α = 1.42), a pattern characteristic of persistent, amoeboid invasion [40]. HURP silencing converted this to sub-diffusive behavior (α = 0.97), effectively abolishing the super-diffusive motility signature. In contrast, MCF10A cells shifted only from sub-diffusive to diffusive motion upon HURP loss, and T47D cells remained sub-diffusive under both conditions. This hierarchy of migration phenotypes mirrors the hierarchy of mitotic dependencies, reinforcing the link between HURP function and metastatic potential.

One possible mechanism connecting HURP to super-diffusive amoeboid motility operates through its role in stabilizing microtubules. HURP could support the rapid cytoskeletal remodeling required for amoeboid movement and super-diffusive migration, and its loss could trap MDA-MB-231 cells in less invasive migration states. Alternatively, HURP could operate through the Aurora-A/centrosome axis. Aurora-A activity at the centrosome during interphase promotes centrosome polarization toward the leading edge, which is required for persistent directional movement and depends on intact TPX2–Aurora-A interaction [24]. Disruption of HURP may impair interphase Aurora-A activity at centrosomes, compromising centrosome polarity and destabilizing the persistent migration front that underlies super-diffusive motility in aggressive cancer cells. Loss of this polarity would be expected to convert persistent, directionally biased movement to constrained, sub-diffusive behavior—precisely what we observe. This interpretation also reconciles the apparent paradox between HURP’s role in microtubule stabilization and amoeboid migration, which is generally considered less microtubule-dependent than mesenchymal movement: the primary effect is centrosomal rather than cytoskeletal.

The conversion of MDA-MB-231 cells from super-diffusive to sub-diffusive migration upon HURP loss supports HURP as a regulator of migration plasticity—the ability of cells to switch between invasive modes [44]—a property that is critical for navigating heterogeneous microenvironments during metastasis.

### 3.6. Implications for Cancer Cell Biology and Therapeutic Strategy

The pronounced sensitivity of MDA-MB-231 cells to HURP loss—encompassing apoptosis, G2/M arrest, spindle collapse, impaired migration, and disrupted spheroid architecture—compared to the comparatively mild effects in non-transformed MCF10A cells positions HURP as a candidate therapeutic target with intrinsic cancer selectivity. This differential vulnerability is consistent with non-oncogene addiction, wherein cancer cells with elevated genomic instability become selectively dependent on regulatory proteins to sustain viability under conditions of chronic replicative stress [3].

A key conceptual question raised by these findings concerns the apparent paradox between HURP overexpression in cancer and the deleterious consequences of its loss. Our data suggest that these are not contradictory: overexpressed HURP in highly proliferating cancer cells likely acts as a mitotic accelerator and a buffer that sustains a permissive level of chromosomal instability (CIN) favorable to clonal evolution, consistent with the “just-right” CIN model [45,46,47]. In this framework, elevated HURP enables cancer cells to tolerate—and selectively exploit—low-level mitotic errors without crossing the threshold of lethal CIN. Importantly, the oncogenic consequences of HURP overexpression are not necessarily restricted to its mitotic role. Non-mitotic functions of HURP, potentially including centrosome regulation, cytoskeletal remodeling during interphase, and Aurora-A-dependent effects on cell polarity and motility, may independently contribute to the migratory and invasive phenotypes observed in highly metastatic cells. These interphase functions remain incompletely characterized and represent an important direction for future investigation. Taken together, this model positions HURP not simply as a mitotic protein whose overexpression drives CIN, but as a multifunctional oncogenic effector whose context-dependent activities span cell division and cell migration.

HURP’s integration within the Aurora-A/TPX2 oncogenic complex [48] further situates it within a clinically validated therapeutic axis: Aurora-A inhibition by alisertib is currently under Phase II clinical investigation in metastatic breast cancer (ALISCA-Breast1, NCT06369285; initiated November 2024), reinforcing the relevance of disrupting this complex in aggressive disease (Puma Biotechnology, Inc., Los Angeles, CA, USA). Our findings suggest that co-targeting HURP alongside Aurora-A may achieve greater selectivity for TNBC by exploiting the compounded dependency of metastatic cells on spindle-associated protein complexes for both mitotic survival and migratory behavior.

### 3.7. Future Directions and Mechanistic Questions

While our study establishes HURP’s multifaceted role in cancer progression, several mechanistic questions remain. The precise molecular mechanisms underlying HURP’s cytoplasmic redistribution in MDA-MB-231 metastatic cells—including whether Aurora-A-dependent hyperphosphorylation, altered importin-β affinity, or other post-translational modifications are responsible—remain to be defined. The potential non-mitotic functions of HURP and their direct contribution to migratory behavior also warrant investigation. Additionally, the apparent compensatory mechanisms that enable non-transformed cells to tolerate HURP loss could reveal alternative therapeutic targets or resistance mechanisms.

The integration of single-cell approaches with our current model system could provide insights into the heterogeneity of HURP-dependent responses within cancer cell populations and reveal how asymmetric mitoses contribute to the generation of migratory subclones.

## 4. Materials and Methods

### 4.1. Cell Culture and Synchronization Conditions

MCF10A (ATCC CRL-10317), T47D (ATCC HTB-133), and MDA-MB-231 (ATCC HTB-26) cell lines were originally sourced from the American Type Culture Collection (ATCC, Manassas, VA, USA). MCF10A was kindly provided by Dr. Clément Thomas (Luxembourg Institute of Health, Luxembourg), and T47D and MDA-MB-231 were kindly provided by Dr. Evelyne Friederich (Luxembourg Centre for Systems Biomedicine, University of Luxembourg, Esch-sur-Alzette, Luxembourg). MCF10A is a non-tumorigenic human mammary epithelial cell line established from the breast tissue of a patient with fibrocystic disease. T47D was derived from the pleural effusion of a patient with infiltrating ductal carcinoma of the breast (ER+, PR+, HER2−). MDA-MB-231 was derived from the pleural effusion of a patient with metastatic adenocarcinoma of the breast (ER−, PR−, HER2−). All cell lines were authenticated by short tandem repeat (STR) profiling and tested negative for mycoplasma contamination prior to use.

MCF10A cells were cultured in DMEM/F12 medium (PAN-Biotech, Aidenbach, Germany) supplemented with 5% horse serum (Lonza, Basel, Switzerland), 20 ng/mL epidermal growth factor (Sigma-Aldrich, St. Louis, MO, USA), 0.5 μg/mL hydrocortisone (Sigma-Aldrich), 100 ng/mL cholera toxin (Sigma-Aldrich), 10 μg/mL insulin (Sigma-Aldrich), 100 U/mL penicillin and 100 μg/mL streptomycin (Lonza). T47D and MDA-MB-231 breast cancer cell lines were maintained in RPMI-1640 medium (Biosera, Nuaillé, France) supplemented with 10% fetal bovine serum (PAN-Biotech), 2 mM L-glutamine (Lonza), 100 U/mL penicillin, and 100 μg/mL streptomycin (Lonza). All cell lines were incubated at 37 °C in a humidified atmosphere containing 5% CO_2_.

For metaphase synchronization, cells were treated with 50 ng/mL nocodazole (Sigma-Aldrich) for 6 h, and, after thorough washing with PBS (1–3 times), cells were released into growth medium containing 10 μg/mL MG132 (Calbiochem, La Jolla, CA, USA) for 2 h.

### 4.2. siRNA Transfection and HURP Silencing

Cells were transfected with either control non-targeting siRNA (5′-UUCUCCGAACGUGUCACGUTT-3′) (Eurofins Genomics) or 50 nM of HURP-specific siRNA (5′-TGACTCGATCAGCTACTCA-3′) (Eurofins Genomics) using JetPRIME transfection reagent (Polyplus, Strasbourg, France) according to the manufacturer’s instructions. Briefly, cells were seeded at 60–70% confluence in antibiotic-free medium 24 h prior to transfection. Transfection efficiency was assessed 48 h post-transfection by Western blot analysis.

### 4.3. Protein Extraction and Western Blot Analysis

Mitotic extracts were obtained by treating cells with nocodazole (100 ng/mL) for 16 h, followed by mechanical shake-off of rounded mitotic cells. Mitotic shake-off after nocodazole treatment provides a highly enriched, but not entirely homogeneous, mitotic cell population; the typical mitotic index under these conditions is 85–95%, as determined by visual inspection. Total protein extracts were prepared by lysing cells in RIPA buffer (50 mM Tris-HCl pH 8, 150 mM NaCl, 1% NP-40, 50 mM sodium orthovanadate, 10 mM sodium fluoride, 0.1% *v*/*v* NP-40, 1 mM PMSF) supplemented with protease inhibitor cocktail (Roche, Basel, Switzerland) for 30 min at 4 °C, followed by centrifugation at 17,900 *g* for 30 min at 4 °C.

Protein concentrations were determined using the Bradford assay (Bio-Rad, Hercules, CA, USA). Equal amounts of protein (20 μg) were separated by SDS-polyacrylamide gradient gels (6–12%) and transferred to nitrocellulose membranes. Membranes were incubated overnight at 4 °C with primary antibodies against HURP (1:1000; rabbit polyclonal antiserum DHL5) [7] and α-tubulin (1:1000; mouse monoclonal antibody, Santa-Cruz Biotechnology, Dallas, TX, USA). Membranes were washed with PBS/0.5% *v*/*v* Tween-20 (Sigma) pH 7.4 and probed with the appropriate rabbit anti-mouse IgG H&L (HRP) (1:20,000; Abcam, Cambridge, UK) and goat anti-rabbit IgG H&L (HRP) (1:20,000; Abcam, Cambridge, UK) secondary antibodies for 1 h at 4 °C. Relative protein expression was quantified using Image Lab (Bio-Rad, Hercules, CA, USA).

### 4.4. Cell-Cycle Analysis by Flow Cytometry

Cells were fixed in 70% ice-cold ethanol for 1 h at 4 °C, centrifuged at 800 *g* for 5 min at 4 °C, washed twice with ice-cold PBS pH 7.4, treated with 100 μg/mL RNase A (Thermo Fisher Scientific, Waltham, MA, USA) to remove RNA, and stained with 50 μg/mL propidium iodide (Invitrogen, Waltham, MA, USA) in PBS pH 7.4 for 40 min in the dark at RT. Flow cytometry was performed with an 8-color fluorescence-activated cell sorting (FACS) Canto II flow cytometer (BD Biosciences, Franklin Lakes, NJ, USA), and the BD FACSDiva™ software version 9.0 was used for data acquisition. A minimum of 10,000 events were collected for each condition. Data were processed using FlowJo V10 software (BD Biosciences).

### 4.5. Immunofluorescence Microscopy

Cells were grown on No. 1.5 glass coverslips or ibidi μ-slide 8-well plates (Ibidi GmbH, Gräfelfing, Germany), fixed in 4% paraformaldehyde/PHEM (60 mM PIPES, 25 mM HEPES, 10 mM EGTA, 2 mM MgCl2) pH 6.9 for 12 min, and permeabilized in PBS/0.1% *v*/*v* Triton X-100 pH 7.4 for 5 min at room temperature (RT). Fixed samples were blocked in PBS/5% *w*/*v* BSA pH 7.4 for 20 min at RT and incubated with primary antibodies against HURP (1:200; chicken polyclonal antibody, Abcam, Cambridge, UK, or rabbit polyclonal DHL5 [7], NuMA (1:200; rabbit monoclonal antibody, Abcam, Cambridge, UK), Aurora-A (1:1000; mouse monoclonal antibody, BD Transduction Laboratories, San Jose, CA, USA), TPX2 (1:100; rabbit polyclonal antibody, Atlas Antibodies, Stockholm, Sweden), α-tubulin (1:1000; mouse monoclonal antibody, Santa Cruz Biotechnology, or 1:200; rabbit polyclonal antibody, Proteintech, Rosemont, IL, USA), BubR1 (1:500; mouse monoclonal, Millipore, Burlington, MA, USA), and ACA (1:1000; human polyclonal) overnight at 4 °C. Cells were washed with PBS pH 7.4, incubated with the appropriate CF^®^488A, CF^®^568, or CF^®^640R secondary antibodies (1:1000; Biotium, Fremont, CA, USA) for 30 min at RT^o^C. DNA was counterstained with Hoechst (10 μg/mL; Life Technologies, Carlsbad, CA, USA) or SPY555-DNA (1 μM; Spirochrome, Stein am Rhein, Switzerland). Following immunostaining, coverslips were mounted in Mowiol 4–88 mounting medium (Applichem GmbH, Darmstadt, Germany).

Imaging was performed on a customized Andor Revolution Spinning Disk Confocal system (Yokogawa CSU-X1; Yokogawa, Tokyo, Japan) built around an Olympus IX81 (Olympus, Shinjuku, Tokyo, Japan) with a 60 × 1.42 NA oil lens (UPlanXApo; Olympus, Shinjuku, Tokyo, Japan) and a digital camera (Andor Zyla 4.2 sCMOS; Andor Technology Ltd., Belfast, Northern Ireland) (CIBIT-Bioimaging Facility, MBG-DUTH). The system was controlled by Andor IQ3.6.5 software (Andor Technology). Images were acquired as z-stacks with optical sections selected at intervals of 0.1, 0.5, or 1 μm throughout the cell volume, according to experimental needs.

### 4.6. Image and Spindle Analysis

Image analysis was performed using ImageJ Version 1.54r (National Institutes of Health, Bethesda, MD, USA) and MATLAB Version R2026a (The MathWorks, Inc., Natick, MA, USA), for which image-processing macros were developed. All immunofluorescence images presented here are the maximum-intensity projections of z-stacks, without background subtraction. The mean fluorescence intensities on the metaphase spindle of HURP, NuMA, TPX2, and Aurora-A were measured by drawing an ellipse enclosing the spindle in the tubulin channel. Background was subtracted using a fixed-size circle drawn outside the cell where mean fluorescence-intensity values were measured. For all the proteins of interest, the fluorescence-intensity plots (%) presented here are normalized to tubulin intensity.

Pole-to-pole intensity profile plot profiles were measured by drawing a line with a width of 60 pixels (3 μm) in the tubulin channel. HURP full-width at half-maximum and half-spindle lengths were measured in the plot profiles. Briefly, the center of the metaphase spindle was determined by fitting a Gaussian curve to the DNA plot profile. Half-spindle lengths were determined as the distances from the center to the left and right poles. The points with the highest HURP intensity in the left and right half-spindles were used as references for measuring the full widths at half maximum (FWHMs) (see Figure S1B). For protein distribution analysis, the Area Under Curve (AUC) was quantified from the normalized plot profile of each spindle. To average the plot profiles from multiple spindles, the plot profile of each spindle was normalized and rescaled using GraphPad Prism 8.0 (GraphPad Software, La Jolla, CA, USA) to account for differences in spindle length.

For spindle orientation analysis, the (x, y, z) coordinates of each pole were measured in the tubulin channel. The angle of the metaphase spindle relative to the growth surface was measured using the equation $\omega =arctan(\frac{z_{distance}}{{xy}_{distance}})$ [49], where $z_{distance}$ represents the distance between the poles in the *z*-axis and ${xy}_{distance}$ represents the distance between the poles in the z-projected image. The spindle long-axis length is represented as the three-dimensional distance between the poles (see Figure S1C). Only bipolar spindles were included in the analysis. To measure the architectural features of the spindles, the Spindle3D [37] plugin in ImageJ was used.

In order to quantify the spindle organization, mitotic spindles were classified into 2 categories: normal and abnormal. Spindles that were characterized by symmetry, well-organized microtubules, and aligned and congressed chromosomes on the metaphase plate were defined as normal. On the other hand, abnormal spindles were classified into 2 extra categories: misaligned and multipolar. Spindles that were characterized by asymmetry, as well as misaligned and uncondensed chromosomes, were defined as misaligned. Lastly, spindles with poorly organized microtubules having three or more poles and uncondensed chromosomes were defined as multipolar.

### 4.7. Kinetochore Linking

Prior to kinetochore linking, images were deconvolved and corrected for chromatic aberrations using Huygens (Scientific Volume Imaging, Hilversum, The Netherlands). Chromatic aberrations were corrected using a correction template generated from images of fluorescent beads (Thermo Fisher Scientific, Waltham, MA, USA; Cat. Number: T7284, TetraSpeck™ beads, 0.1 μm).

Kinetochore linking was performed in three-dimensional space. First, metaphase plates were rotated such that their short axis aligned with the *x*-axis of the image. Kinetochores were then detected and segmented using a multi-scale LoG detector applied to the ACA channel. Based on these segmentations, the centroid coordinates and mean fluorescence intensities of ACA and BubR1 were extracted for each detected kinetochore (see Figure S5E).

Sister kinetochores were linked by generating a two-frame pseudo-timelapse sequence and applying the linear assignment problem algorithm to identify the optimal linking solution [50]. A custom cost function was used, incorporating the maximum and minimum distances between the source and target kinetochores, their *z*-axis distance, the angle of the linking vector relative to the *x*-axis, and differences in fluorescence intensity. All generated links were subsequently reviewed manually in ImageJ, and only biologically plausible links were retained for statistical analysis.

### 4.8. Scratch Wound-Healing Assays

Cell migration was assessed using scratch wound-healing assays performed in the presence or absence of mitomycin-C (10 μg/mL for 2 h prior to imaging, Cayman Chemical, Ann Arbor, MI, USA) to distinguish migration from proliferation. Confluent monolayers in ibidi μ-slide 8-well plates (Ibidi GmbH, Gräfelfing, Germany) were scratched using sterile pipette tips, washed with PBS pH 7.4, and monitored at 10 min intervals, for a total of 108 frames (18 h). Imaging was performed on a customized Andor Revolution Spinning Disk Confocal system (Yokogawa CSU-X1; Yokogawa, Tokyo, Japan) built around an Olympus IX81 (Olympus, Shinjuku, Tokyo, Japan) with 10 × 0.3 NA air lens (UPlanFL N; Olympus, Shinjuku, Tokyo, Japan) and a digital camera (Andor Ixon Ultra 897 EMCCD; Andor Technology Ltd., Belfast, Northern Ireland, UK), inside a humidified environmental chamber at 37 °C with 5% CO_2_ (CIBIT-Bioimaging Facility, MBG-DUTH), using phase-contrast microscopy.

ImageJ and the Trainable Weka Segmentation plugin [51] were used to train and then apply a 2-class classifier to the image series. Pixels in each frame of the time series were classified as either foreground (cells) or background (scratch gap). Classified time series were then loaded into MATLAB for post-processing. Initially, each frame was processed to close gaps and correct classification artifacts, and the area corresponding to the gap was measured. To measure the “front velocity”, we modeled the gap as a box with an area equal to Area = Width × Height. Assuming that the gap is much longer than the field of view, i.e., cells do not enter the gap area from below or above the gap, the height (H) remains constant and only the width (W) changes over time. Therefore, we have

(1) $$\frac{dA}{dt}=\frac{dW}{dt}\times H+\frac{dH}{dt}\times W\Rightarrow \;\frac{dA}{dt}=\frac{dW}{dt}\times H\;$$

Moreover, assuming that the two cell fronts have the same velocity (V) we get

(2) $$\frac{dW}{dt}=-2\times V\;$$

Finally, combining Equations (1) and (2), we get

(3) $$\frac{dA}{dt}=\frac{dW}{dt}\times H\Rightarrow \frac{dA}{dt}=-2\times V\times H\Rightarrow V=-\frac{dA}{dt}\times \frac{1}{2\times H}$$

where *V* represents the “front velocity”.

To determine $\frac{dA}{dt}$, we fitted the gap area measurements within a 1 h interval (6 frames) using a linear model (y = λχ + β). The slope of the fitted line is equal to $\lambda \;=\;\frac{dA}{dt}$. Therefore, using Equation (3), we measured the average “front velocity” over 1 h intervals.

### 4.9. Single-Cell Tracking and Migration Analysis

For the single-cell tracking assay, cells were seeded in ibidi μ-slide 8-well plates (Ibidi GmbH, Gräfelfing, Germany). Twenty-four hours before imaging, the growth medium was replaced with fresh medium containing 1 μΜ SiR-DNA (Spirochrome, Stein am Rhein, Switzerland) and incubated overnight. Live imaging was performed on a customized Andor Revolution Spinning Disk Confocal system (Yokogawa CSU-X1; Yokogawa, Tokyo, Japan). Cells were monitored with a 10 min time interval between frames, for a total of 108 frames (18 h), using a 638 nm laser.

Single-cell tracking was performed using the TrackMate plugin [52,53] in ImageJ. Briefly, the LoG detector was used to identify individual cells with sigma values of 20 μm, 13 μm, and 14 μm for MCF10A, T47D, and MDA-MB-231, respectively. The quality threshold was set to 8 for all cell lines. For frame-to-frame linking, a maximum distance of 10 μm was set, with a maximum gap of 2 frames and a maximum gap distance of 20 μm. For detecting cell divisions, the object-splitting option was utilized, with a maximum splitting distance of 12 μm, and the maximum-intensity difference between splitting cells as a penalty feature. The TrackScheme tool was then used to correct and complete tracks. The whole track, including edge features, was exported for further statistical analysis. Only tracks with a duration greater than 940 min were used and presented.

Square displacement was calculated using a custom-built MATLAB script utilizing TrackMate’s “importTrackMateTracks” function and the equation: $r^{2}(t)\;=\;(x_{t}-x_{0}{)}^{2\;}+(y_{t}-y_{0}{)}^{2\;}$, where $\left(x_{0},y_{0}\right)$ and $\left(x_{t},y_{t}\right)$ represent the position coordinates at time t=0 and t=t, respectively. The mean square displacement (MSD) was calculated by averaging across all cells. MSD over time was plotted on a log-log scale and fitted with an exponential $x^{2}\propto t^{\alpha }$, where exponent α represents the slope of the line. Values of α < 1, α = 1, and α > 1 indicate sub-diffusive, diffusive, and super-diffusive motion, respectively [54,55].

### 4.10. MDA-MB-231 Spheroid Formation

MDA-MB-231 cells were plated on a 6-well plate (SPL, Pocheon, Gyeonggi-do, Republic of Korea) and cultured in two dimensions (40,000 cells per well). When 30% confluence was reached, RNA transfection (50 nM siRNA duplexes) was performed according to the manufacturer’s instructions (jetPRIME^®^ Versatile DNA/siRNA transfection reagent, 101000015, Polyplus^®^, France), and cells were incubated for 6 h (5% CO_2_, 95% humidity, 37 °C). Cells were detached from their growing surface via trypsinization (5 min at 37 °C) and centrifuged at 500 *g* for 5 min. Cell density was calculated, and cell suspensions were prepared, so that 1000 cells were seeded per well in 50 μL RNA-containing medium. Cells were seeded onto a Nunclon™ Sphera™ 96-well U-shaped-bottom microplate (Thermo Fisher Scientific, Waltham, MA, USA) and centrifuged for 10 min at 200 *g*. Following the initial centrifugation, 50 μL of rBM (Matrigel)-containing medium was added so that each well contained 2% matrix (Matrigel^®^ Matrix, Corning^®^, Corning, NY, USA), and plates were centrifuged again for 10 min at 500 *g*. Plates were placed in the incubator (5% CO_2_, 95% humidity, 37 °C) in order to enable spheroid formation. Spheroids were harvested 48 h later and transferred into U-bottom 2 mL Eppendorf tubes.

### 4.11. Two-Dimensional Spheroid Imaging and Analysis

Spheroids were imaged using a conventional phase-contrast microscope (Oxion Inverso, Euromex Microscopen B.V., Arnhem, The Netherlands) equipped with a 4 × objective (LPlan 4×/0.13; Euromex) and a CMOS camera (CMEX-3 Pro; Euromex) at day 2. To quantify the two-dimensional geometric features of spheroids, the CATS plug-in [56] in ImageJ was used to segment spheroids from background, by first training and then applying a 2-class pixel-based classifier. Binary masks were then imported into MATLAB for processing and extraction of spheroid geometric properties, as shown in Figure 7.

### 4.12. Spheroid Immunofluorescence

Fixation was performed in 4% paraformaldehyde/PHEM (60 mM PIPES, 25 mM HEPES, 10 mM EGTA, 2 mM MgCl2) (Applichem GmbH, Darmstadt, Germany) at pH 6.9 for 10 min, permeabilization in 0.3% Triton X-100 (TR0444, Scharlab S.L., Barcelona, Spain) in PBS 1× pH 7.4 for 15 min, and blocking was performed in “blocking buffer” containing 0.2% Triton X-100 and 5% BSA (P06-139310, PAN-Biotech GmbH, Aidenbach, Germany) in PBS 1× for 1 h at RT. Incubation with primary antibodies against HURP (1:1000; rabbit polyclonal DHL5 [7]) and tubulin (1:1000; mouse monoclonal antibody, Santa Cruz Biotechnology, USA) was performed in blocking buffer at 37 °C overnight. Incubation with secondary antibodies (1:1000 CF488A goat anti-mouse IgG (H + L) [Biotium], 1:1000 CF640R donkey anti-rabbit IgG (H + L) [Biotium]), and DNA dye (1:1000 SPY555-DNA [SC201, Spirochrome]) was performed in blocking buffer at 37 °C overnight. Spheroids were stored at 4 °C in PBS 1× until imaging. Prior to imaging, spheroids were mounted in 1% low-melting agarose (732893, FMC BioProducts, Philadelphia, PA, USA) in PBS 1× and then aspirated into fluorinated ethylene propylene (FEP) tubes (Fluorotherm™, Parsippany, NJ, USA).

### 4.13. Single-Plane Illumination Microscopy (SPIM) Imaging and 3D Spheroid Analysis

MDA-MB-231 spheroids were imaged in three dimensions using a single-plane illumination microscope (Ultramicroscope II–Miltenyi Biotec, Bergisch Gladbach, Germany). Samples were illuminated from both sides using only the central light sheet. The thickness of each light sheet was set to 2w = 7.53 μm. Fluorophore excitation was achieved using the 488 nm, 561 nm, or 640 nm laser lines. Fluorescence detection was performed using a 16× (0.8 NA) water-dipping objective (Nikon Instruments Inc., Amstelveen, The Netherlands); emission filters (525/20, 620/60, 680/30); a zoom body with 2× post-magnification, resulting in a total magnification of 28.8×; and an Andor Neo 5.5 sCMOS camera with a pixel size of 6.5 μm (Andor Technology, Belfast, UK). Agarose-embedded spheroids were extruded from the FEP tube and placed in a custom 3D-printed holder, which allowed sample rotation. Spheroids were placed in the imaging cuvette filled with PBS 1× and imaged with a z-step size of 1 μm. Image acquisition was performed via the ImSpectorPro software (Version 7.1.15) (Miltenyi Biotec, Germany). Each spheroid was imaged in two rotational views (0^0^ and 180^0^). Rotation was performed using a step motor (28BYJ-48) driven by an Arduino UNO.

Fusion of the rotational views was performed using Huygens software 25.x version (Scientific Volume Imaging, Hilversum, The Netherlands). Briefly, each rotational view was first deconvolved and then fused using the light-sheet fuser tool.

An intensity-based threshold was set to create spheroid masks and extract geometric features. Initial masks were further processed with morphological operations to fill holes and gaps. The MATLAB function “regionprops3” was used to measure the volume, convex volume, solidity, surface area, and principal-axis lengths. Sphericity was computed using the following equation:

(4) $$Sphericity=\frac{{\left(36\times \pi \times Volume^{2}\right)}^{\frac{1}{3}}}{Surface\;Area}$$

### 4.14. Statistical Analysis

Statistical analysis (Mann–Whitney two-tailed test) and plotting were performed using GraphPad Prism 8.0 (GraphPad Software, La Jolla, CA, USA).

## 5. Conclusions

This study establishes HURP as a multifunctional regulator that coordinates mitotic fidelity, spindle orientation, migration plasticity, and tissue architecture in breast cancer cells. The progressive alteration of HURP functions across the metastatic gradient, combined with the selective vulnerability of aggressive cells to its loss, positions HURP and its associated protein network as promising targets for cancer therapy. These findings define a therapeutically relevant dependency within the Aurora-A/TPX2/NuMA signaling axis and contribute to the growing understanding of how cancer cells co-opt fundamental mitotic machinery to enable tumor progression and metastatic dissemination.

## Data Availability

All supplementary movies are available through the following link: https://zenodo.org/records/20575218 (accessed on 7 June 2026). Original data are available here: https://zenodo.org/records/20575883 (accessed on 7 June 2026).

## Supplementary Materials

The following supporting information can be downloaded at: https://www.mdpi.com/article/10.3390/ijms27135897/s1.
