# Supplementary material for: HURP Silencing Differentially Impacts Spindle Architecture and Metastatic Behavior in Breast Cancer Cell Lines
Source: Int J Mol Sci. 2026 Jun 30;27(13):5897. doi: 10.3390/ijms27135897 (PMC13360948; doi:10.3390/ijms27135897)
Supplement: Supplementary file 1 [file ijms-27-05897-s001.zip › ijms-4393353-supplementary.pdf]

Figure S1

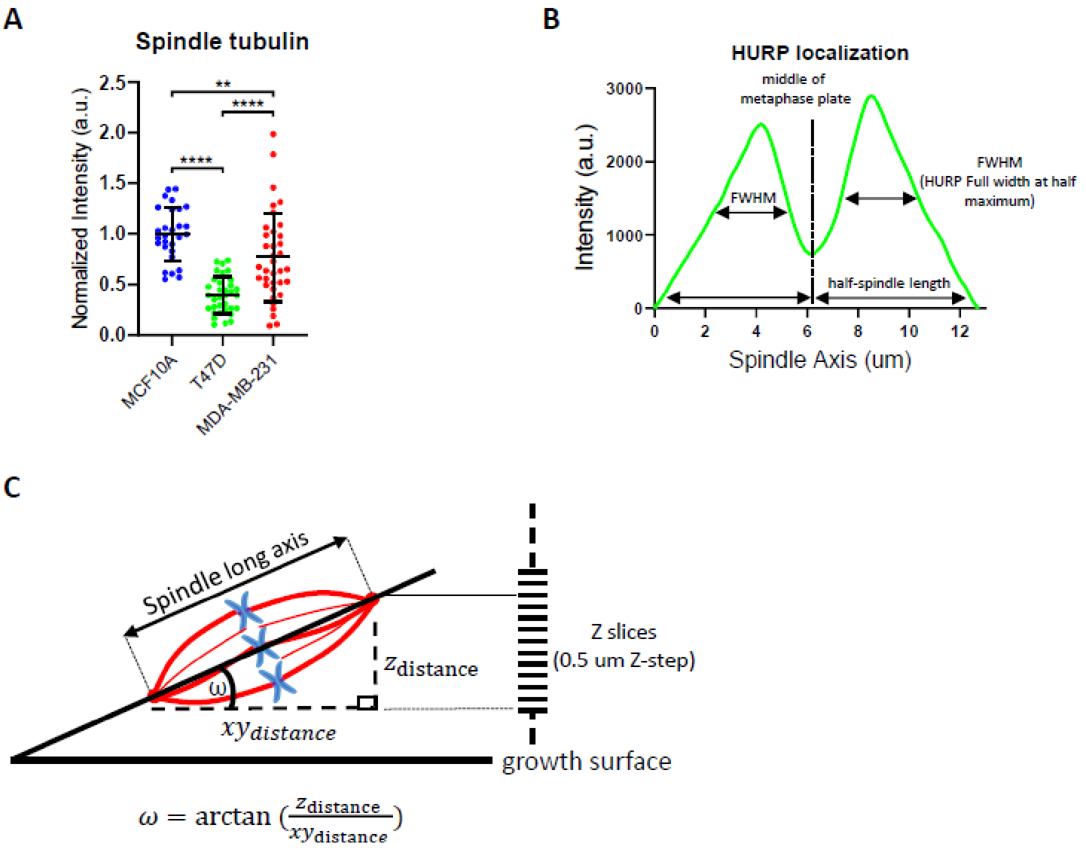

Figure S1. Spindle tubulin quantification, HURP FWHM analysis schematic, and spindle orientation measurement methodology. (A) Scatter plots of normalized spindle tubulin fluorescence intensity (a.u.) in metaphase-arrested MCF10A, T47D, and MDA-MB-231 cells (MCF10A, n = 28; T47D, n = 34; MDA-MB-231, n = 36). $^{**}p < 0.01$; $^{****}p < 0.0001$; two-tailed Mann-Whitney test. Error bars represent SD. (B) Schematic illustrating HURP spatial distribution analysis. HURP full width at half maximum (FWHM) was measured from the HURP pole-to-pole intensity profile. Half-spindle length was determined as the pole-to-midpoint distance derived by fitting a Gaussian curve to the DNA channel. The FWHM-to-half-spindle-length ratio reflects the fraction of the spindle occupied by HURP. (C) Schematic representation of 3D spindle orientation analysis. The spindle angle (ω) relative to the growth substrate is calculated as $\omega = \arctan(z_{distance} / xy_{distance})$ (Toyoshima and Nishida, 2007), where z_distance and xy_distance are the inter-polar distances in the z-axis and in the z-projection, respectively.

Figure S2

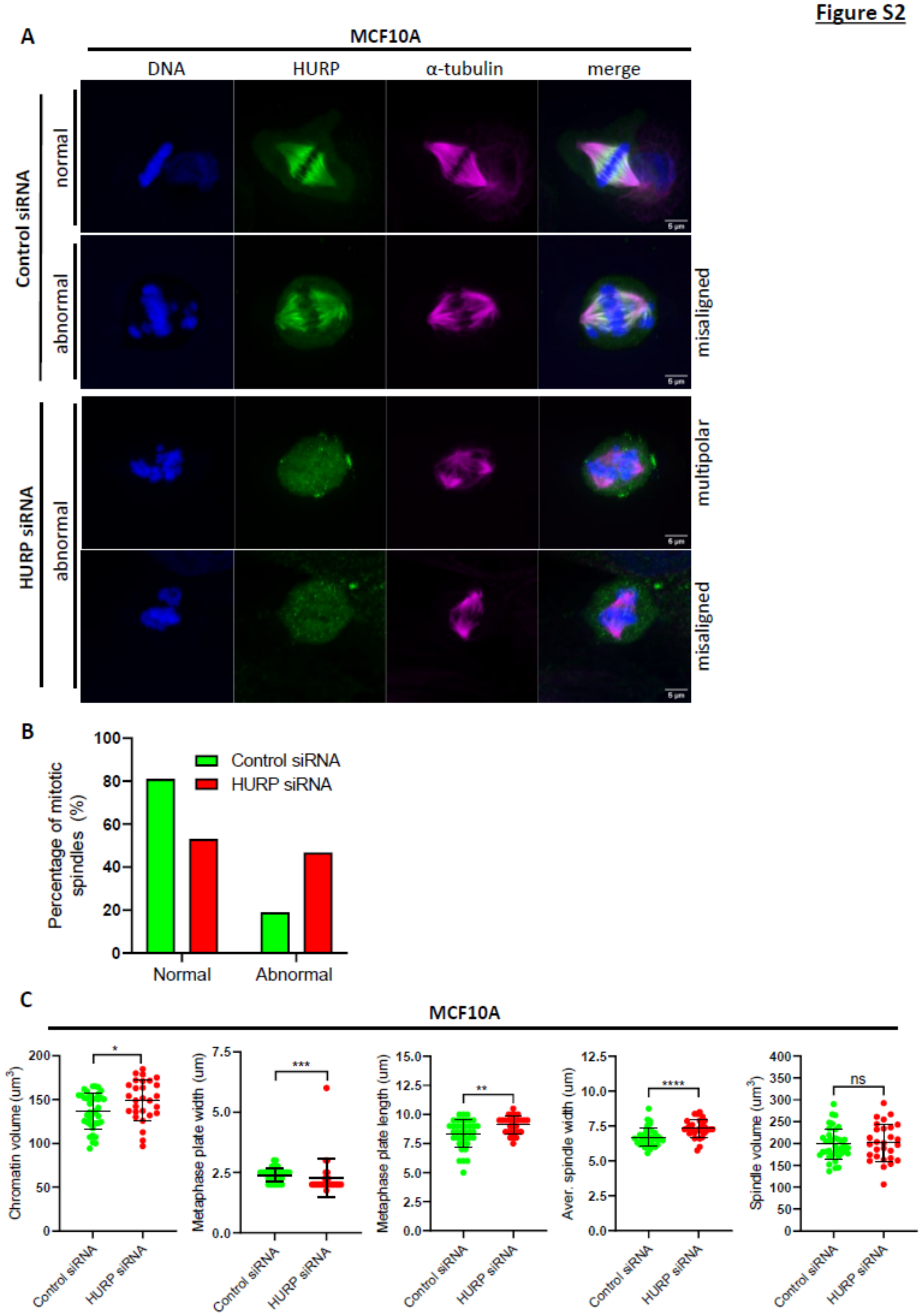

Figure S2. Impact of HURP silencing on spindle architecture in MCF10A cells. (A) Representative immunofluorescence images of metaphase-arrested MCF10A cells treated with control siRNA (upper panels) or HURP siRNA (lower panels). The most frequent phenotypes for each condition are illustrated. Cells were stained for HURP (green), α-tubulin (magenta), and DNA (blue, Hoechst). Scale bars, 5μm. (B) Immunoblot analysis of total protein extracts from control and HURP siRNA-treated MCF10A cells, probed for HURP with α-tubulin as loading control. (C) Quantification of spindle organization phenotypes in metaphase-arrested MCF10A cells following control or HURP siRNA treatment (control siRNA, n = 43; HURP siRNA, n = 32). (D) Scatter plots of 3D spindle architecture parameters measured using the Spindle3D plugin: chromatin volume, metaphase plate width and length, mean spindle width, and spindle volume, in MCF10A cells treated with control or HURP siRNA (Control siRNA, n = 38 cells; HURP siRNA, n = 28 cells;) *: $0.01<p<0.05$; **: $0.001<p<0.01$; ***: $0.0001<p<0.001$; ****: $p<0.0001$, Mann-Whitney test, two-tailed. Error bars represent S.D.

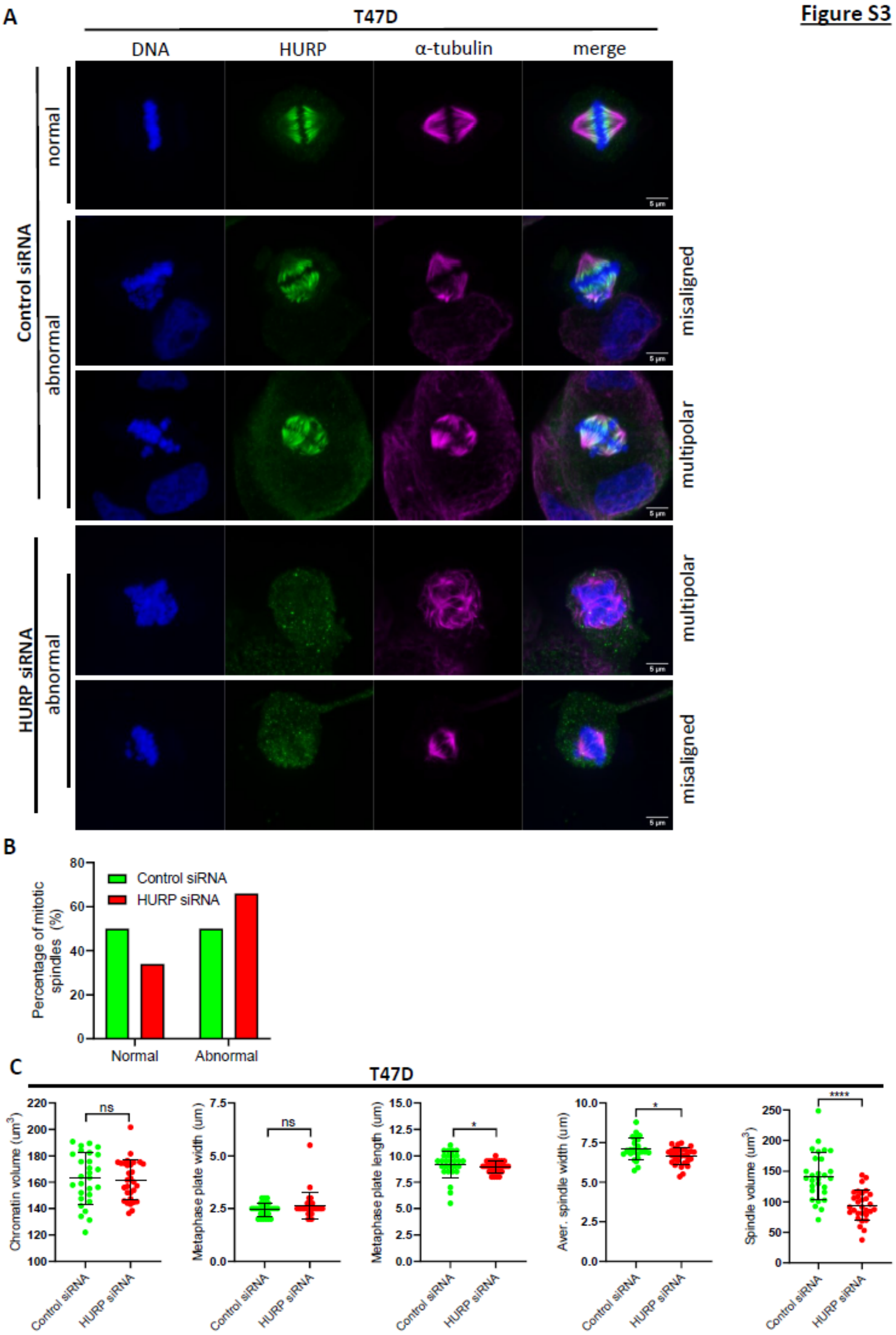

Figure S3. Impact of HURP silencing on spindle architecture in T47D cells. (A) D cells treated with control siRNA (upper panels) or HURP siRNA (lower panels). The most frequent phenotypes for each condition are illustrated. Cells were stained for HURP (green), α-tubulin (magenta), and DNA (blue, Hoechst). Scale bars, 5μm. (B) Immunoblot analysis of total protein extracts from control and HURP siRNA-treated T47D cells, probed for HURP with α-tubulin as loading control. (C) Quantification of spindle organization phenotypes in metaphase-arrested T47D cells following control or HURP siRNA treatment (control siRNA, n = 64; HURP siRNA, n = 44). (D) Scatter plots of 3D spindle architecture parameters in T47D cells treated with control or HURP siRNA (Control siRNA, n = 28 cells; HURP siRNA, n = 30 cells;) *: $0.01<p<0.05$; ****: $p<0.0001$, Mann-Whitney test, two-tailed. Error bars represent S.D.

Figure S4

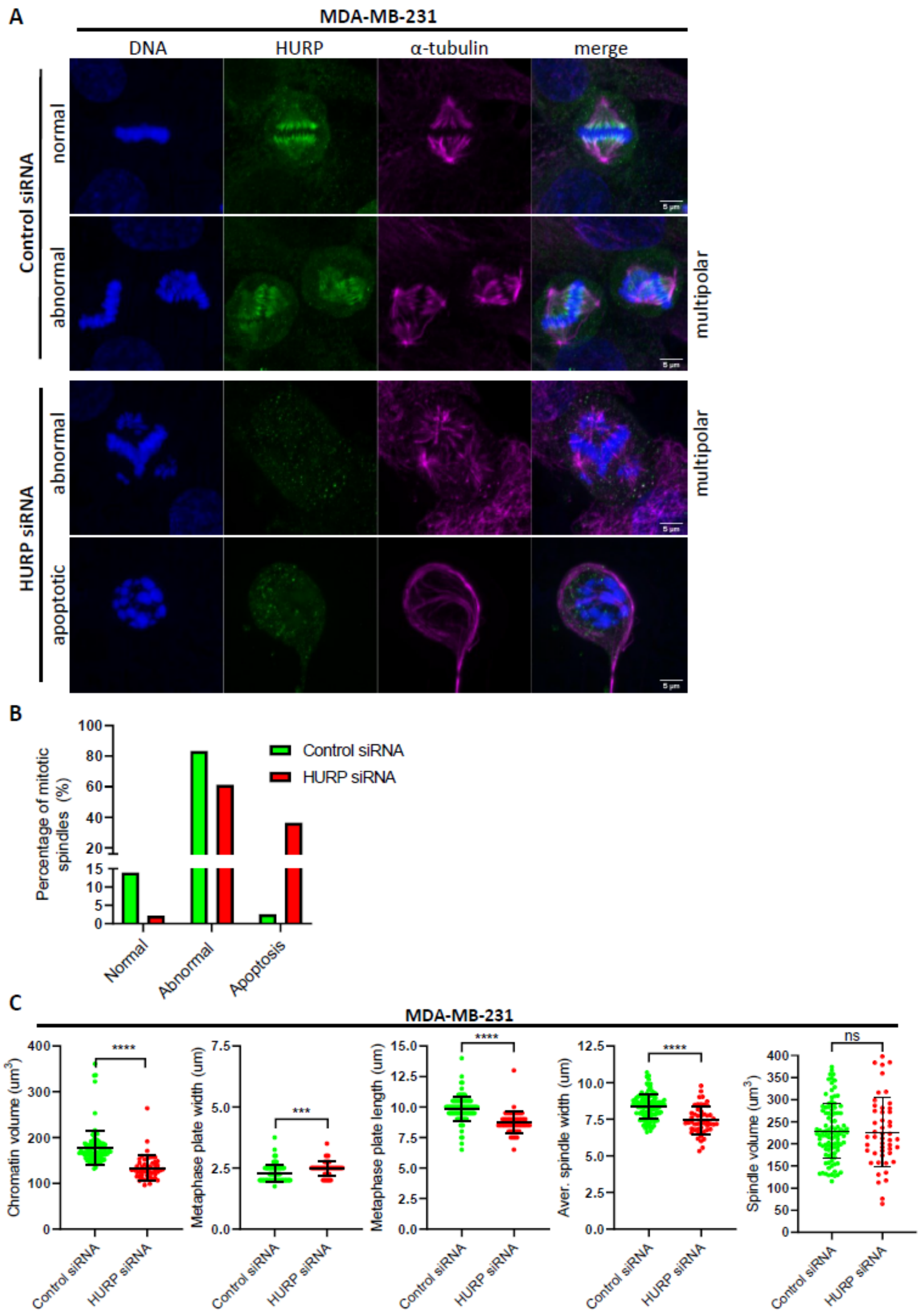

Figure S4. Impact of HURP silencing on spindle architecture in MDA-MB-231 cells. (A) Representative immunofluorescence images of metaphase-arrested MDA-MB-231 cells treated with control siRNA (upper panels) or HURP siRNA (lower panels). The most frequent phenotypes for each condition are illustrated. Cells were stained for HURP (green), α-tubulin (magenta), and DNA (blue, Hoechst). Scale bars, 5μm. (B) Immunoblot analysis of total protein extracts from control and HURP siRNA-treated MDA-MB-231 cells, probed for HURP with α-tubulin as loading control. (C) Quantification of spindle organization phenotypes in metaphase-arrested MDA-MB-231 cells following control or HURP siRNA treatment (control siRNA, n = 193; HURP siRNA, n = 176). (D) Scatter plots of 3D spindle architecture parameters in surviving MDA-MB-231 cells treated with control or HURP siRNA (Control siRNA, n = 103 cells; HURP siRNA, n = 50 cells;) ***: 0.0001<p<0.001; ****: p<0.0001, Mann-Whitney test, two-tailed. Error bars represent S.D

Figure S5

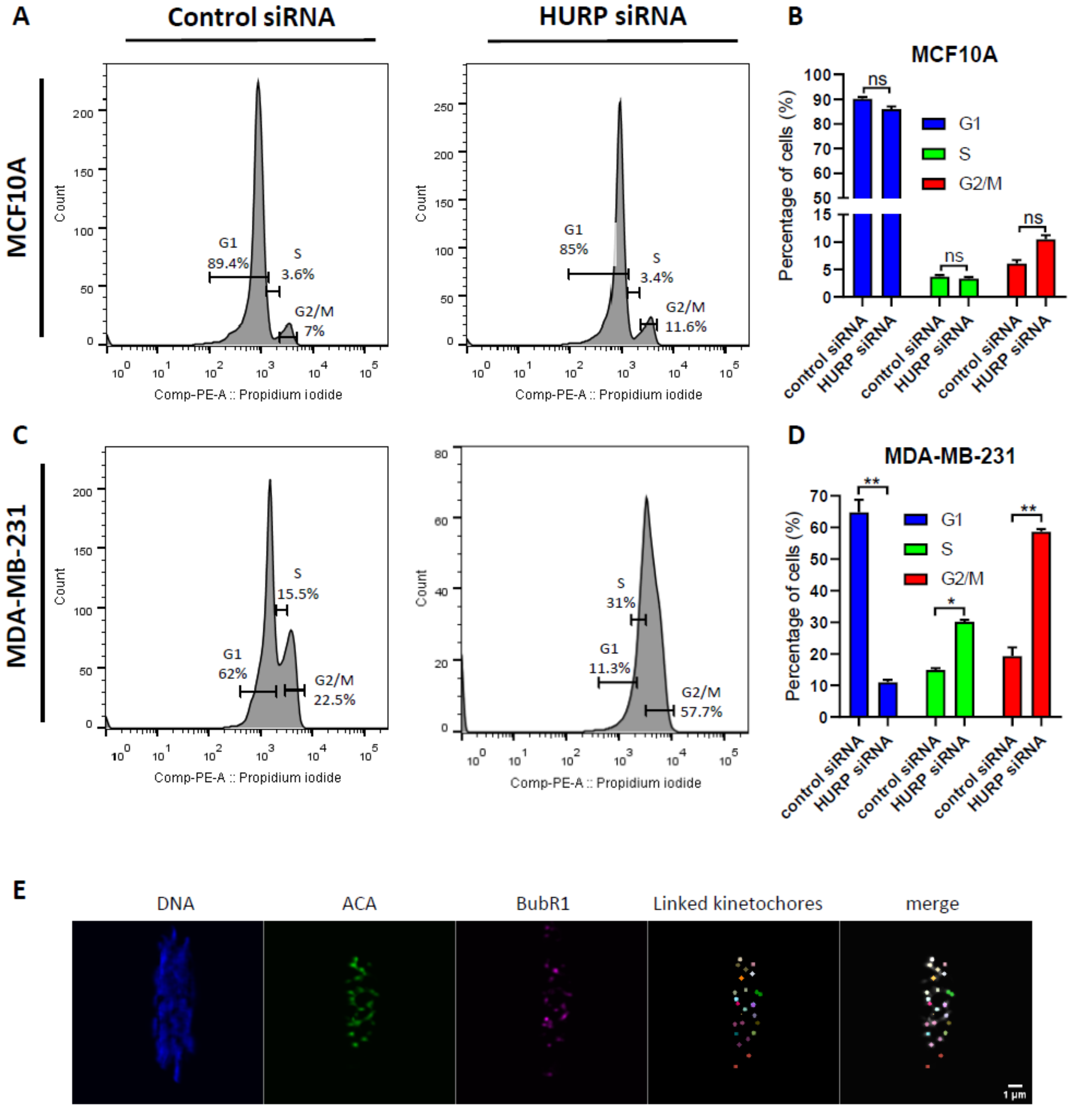

Figure S5. Cell cycle distribution and kinetochore analysis methodology. (A) Representative propidium iodide flow cytometry histograms of cell cycle distribution in MCF10A cells treated with control (left) or HURP siRNA (right). (B) Quantification of cell cycle phase distribution in MCF10A cells (control, N = 4; HURP siRNA, N = 4). ns, not significant; Mann-Whitney test, two-tailed. Error bars represent SD. (C) Representative propidium iodide flow cytometry histograms of cell cycle distribution in MDA-MB-231 cells treated with control or HURP siRNA. Note the reduction in total cell number and the shift in phase distribution. (D) Quantification of cell cycle phase distribution in MDA-MB-231 cells (control, N = 4; HURP siRNA, N = 4). *$p < 0.05$; **$p < 0.01$; Mann-Whitney test, two-tailed. Error bars represent SD. Representative maximum-intensity projection image (10 consecutive z-slices) of an MCF10A cell stained for DNA, ACA (kinetochore marker), and BubR1. Panel 4: segmented and linked sister kinetochore pairs indicated by matching colors. Panel 5: segmented kinetochores overlaid on the ACA channel. Scale bar 1µm. .

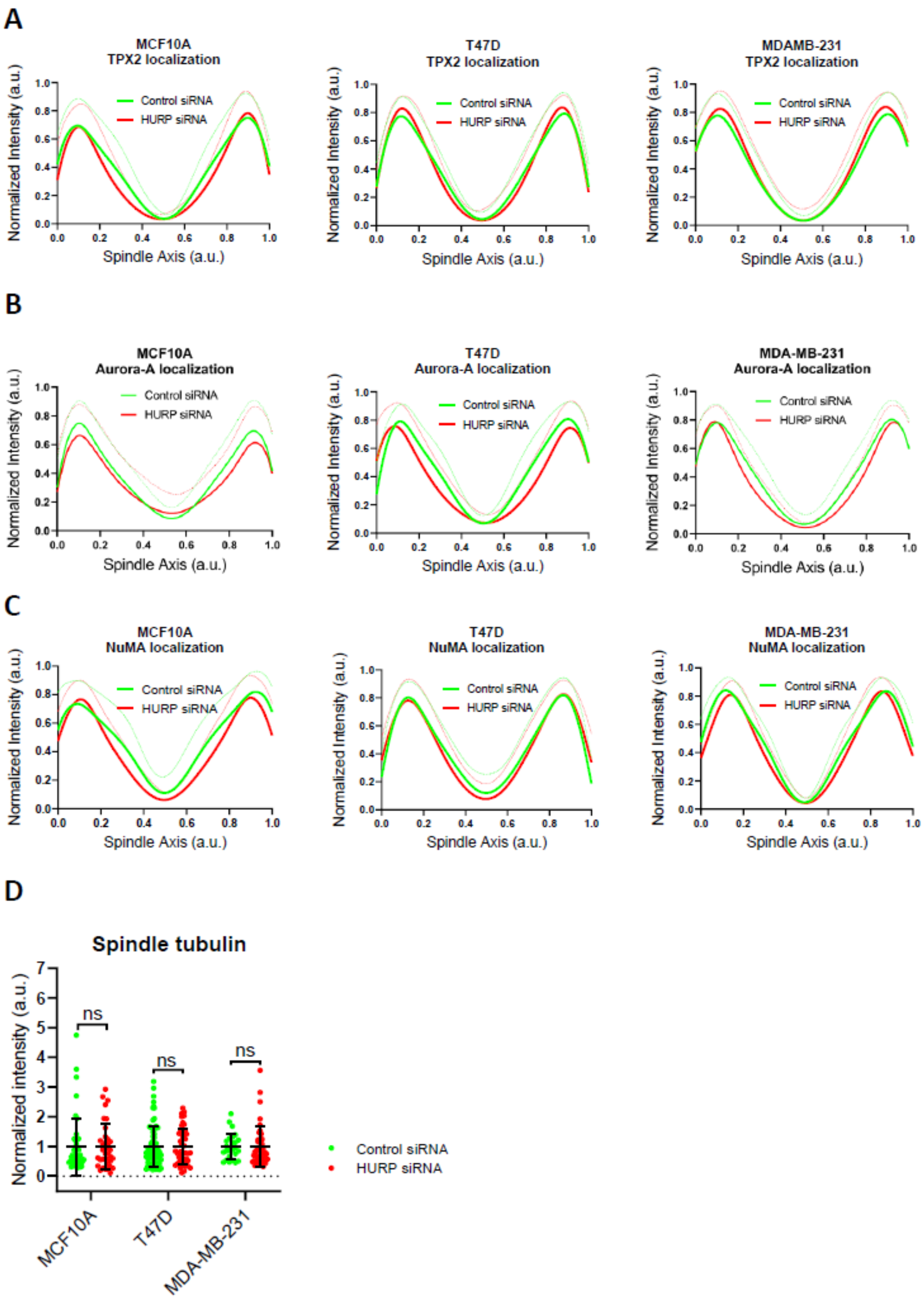

Figure S6. TPX2, Aurora-A and NuMA localization on metaphase spindle and tubulin quantification post HURP silencing. (A) TPX2 intensity profiles along the metaphase spindle axis in MCF10A, T47D and MDA-MB-231 cells treated with control or HURP siRNA. Lines indicate the mean values, whereas dots represent the S.D. (MCF10A: control siRNA, n = 43; HURP siRNA, n = 35;) (T47D: control siRNA, n = 76; HURP siRNA, n = 45;) (MDA-MB-231: control, n = 36; HURP siRNA, n = 52;). (B) Aurora-A intensity profiles along the metaphase spindle axis for each cell line and condition (MCF10A: control, n = 50; HURP siRNA, n = 49;) (T47D: control, n = 38; HURP siRNA, n = 32;) (MDA-MB-231: control, n = 62; HURP siRNA, n = 58;). (C) NuMA intensity profiles along the metaphase spindle axis for each cell line and condition (MCF10A: control, n = 42; HURP siRNA, n = 34;) (T47D: control, n = 82; HURP siRNA, n = 52;) (MDA-MB-231: control, n = 41; HURP siRNA, n = 45;). (D) Scatter plots of spindle-bound α-tubulin fluorescence intensity (a.u.) in control and HURP siRNA-treated cells for each cell line (MCF10A: control, n = 43; HURP siRNA, n = 35; T47D: control, n = 82; HURP siRNA, n = 51; MDA-MB-231: control, n = 41; HURP siRNA, n = 52). All values normalized to the control mean for each cell line. ns: not significant; Mann-Whitney test, two-tailed. Error bars represent S.D.

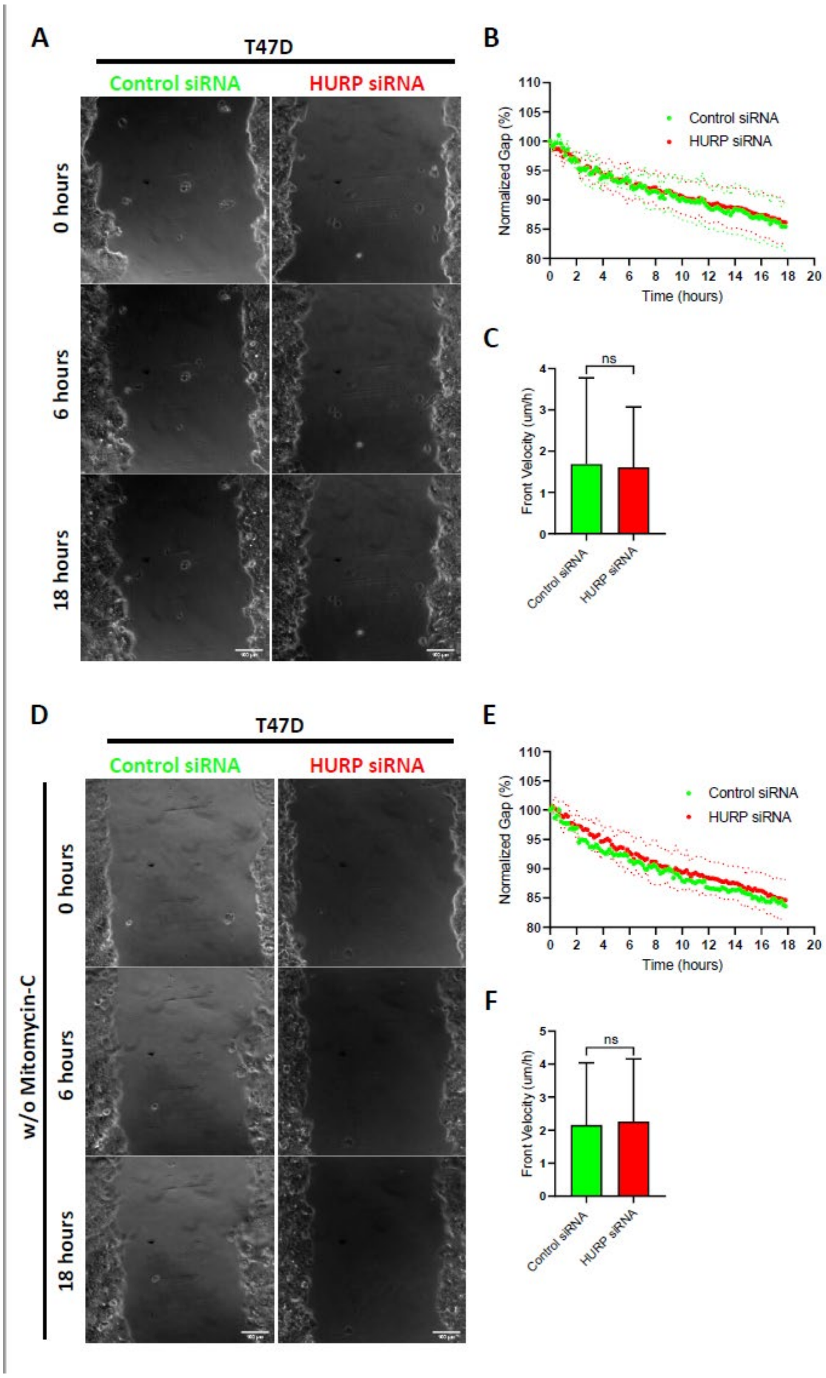

Figure S7. Scratch wound healing assays in T47D cells with and without Mitomycin-C treatment. (A) Representative phase-contrast time-lapse images of scratch assays in Mitomycin-C-treated T47D cells transfected with control (left) or HURP siRNA (right) at 0, 6 and 18 hours. Scale bar, 100µm. (B) Quantification of gap closure over time in Mitomycin-C-treated T47D cells under each condition. (Control, N = 2 fields; HURP siRNA, N = 4 fields;) Small dots represent S.D. (C) Mean front velocity of Mitomycin-C-treated T47D cells under each condition (Control, n = 36 values, N = 2 fields; HURP siRNA, n = 70 values, N = 4 fields;) ns: not significant, Mann-Whitney test, two-tailed. Error bars represent S.D. (D) Representative phase-contrast time-lapse images of scratch assays in T47D cells treated with control (left) or HURP siRNA (right) at 0, 6 and 18 hours. Scale bar, 100µm. (E) Quantification of gap closure over time in T47D cells treated with control or HURP siRNA. (Control, N = 2 fields; HURP siRNA, N = 3 fields;) Small dots represent S.D. (F) Mean front velocity of T47D cells treated with control or HURP siRNA. (Control, n = 36 values, N = 2 fields; HURP siRNA, n = 53 values, N = 3 fields;) ns: not significant, Mann-Whitney test, two-tailed. Error bars represent S.D.

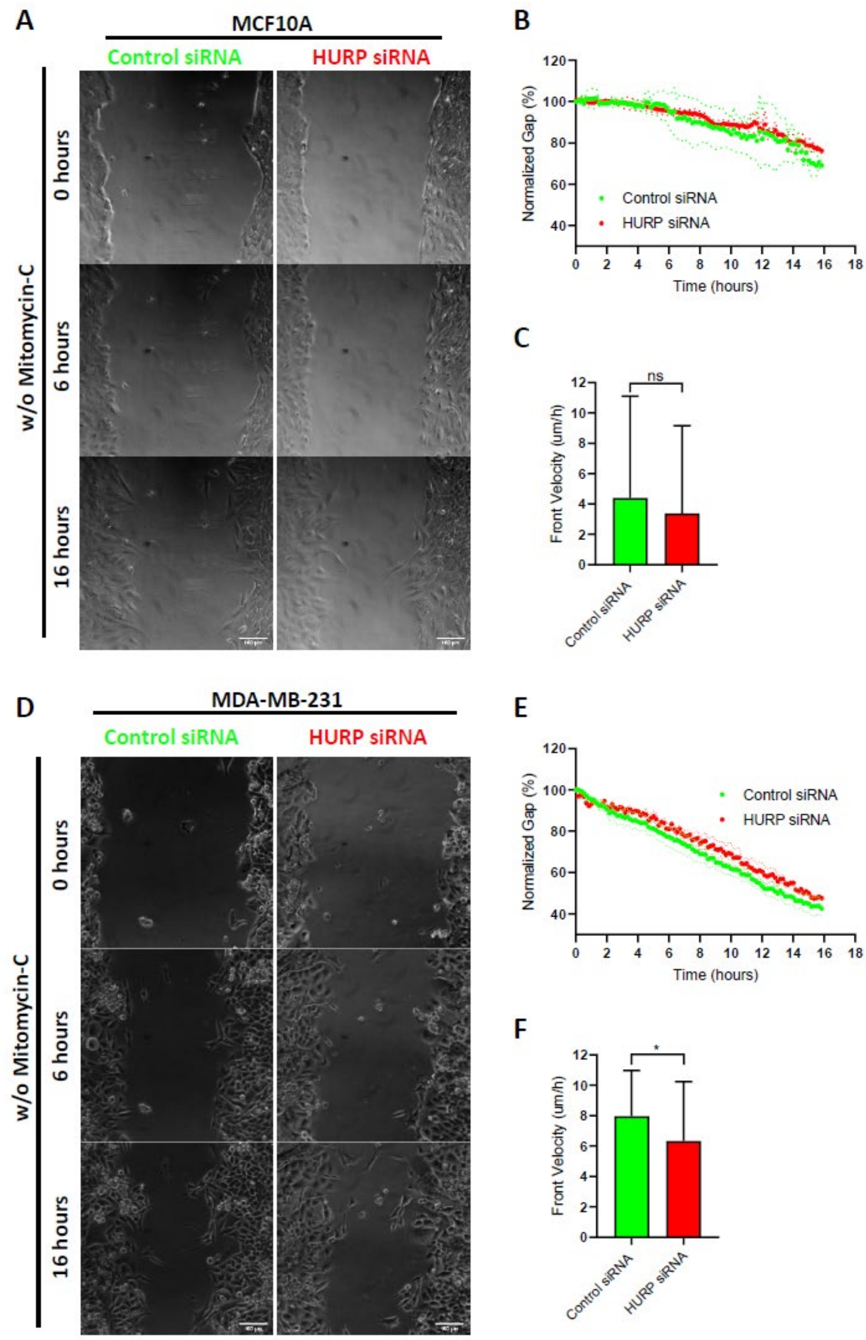

Figure S8. Scratch wound healing assays in MCF10A and MDA-MB-231 cells in the absence of Mitomycin-C. (A) Representative phase-contrast time-lapse images of scratch assays of MCF10A cells treated with control (left) or HURP siRNA (right) at 0, 6 and 16 hours (without Mitomycin-C). Scale bar, 100μm. (B) Quantification of gap closure over time in MCF10A cells treated with control or HURP siRNA. (Control, N = 3 fields; HURP siRNA, N = 3 fields). Small dots represent S.D. (C) Mean front velocity of MCF10A cells under each condition. (Control, n = 54 values, N = 3 fields; HURP siRNA, n = 53 values, N = 3 fields;) ns: not significant, Mann-Whitney test, two-tailed. Error bars represent S.D. (D) Representative phase-contrast time-lapse of scratch assays of MDA-MB-231 cells treated with control (left) or HURP siRNA (right) at 0, 6 and 16 hours). Scale bar, 100μm. (E) Quantification of gap closure over time in MDA-MB-231 cells treated with control or HURP siRNA. (Control siRNA, N = 3 field; HURP siRNA, N = 2 fields) Small dots represent S.D. (F) Mean front velocity of MDA-MB-231 cells under each condition. (Control, n = 48 values, N = 3 fields; HURP siRNA, n = 32 values, N = 2 fields;) *: $0.01<p<0.05$, Mann-Whitney test, two-tailed. Error bars represent S.D.
